# Supplementary material for: Targeting the CD40 costimulatory receptor to improve virotherapy efficacy in diffuse midline gliomas
Source: Cell Rep Med. 2025 Jun 26;6(7):102204. doi: 10.1016/j.xcrm.2025.102204 (PMC12281365; doi:10.1016/j.xcrm.2025.102204)
Supplement: Document S1. Figures S1–S7 [file mmc1.pdf]

Cell Reports Medicine, Volume *6*

Supplemental information

Targeting the CD40 costimulatory receptor to improve virotherapy efficacy in diffuse midline gliomas

Sara Labiano, Javier Marco-Sanz, Iker Ausejo-Mauleon, Virginia Laspidea, Reyes Hernández-Osuna, Marc Garcia-Moure, Daniel de la Nava, Sara Nuin, Marisol Gonzalez-Huarriz, Timothy N. Phoenix, Ibon Tamayo, Marta Zalacain, Andrea Lacalle, Lucía Marrodan, Montserrat Puigdelloses, Irati Hervás-Corpión, Maria C. Ochoa, Noelia Casares, Oren J. Becher, Candelaria Gomez-Manzano, Juan Fueyo, Jaime Gallego Perez-Larraya, Ana Patiño-Garcia, and Marta M. Alonso

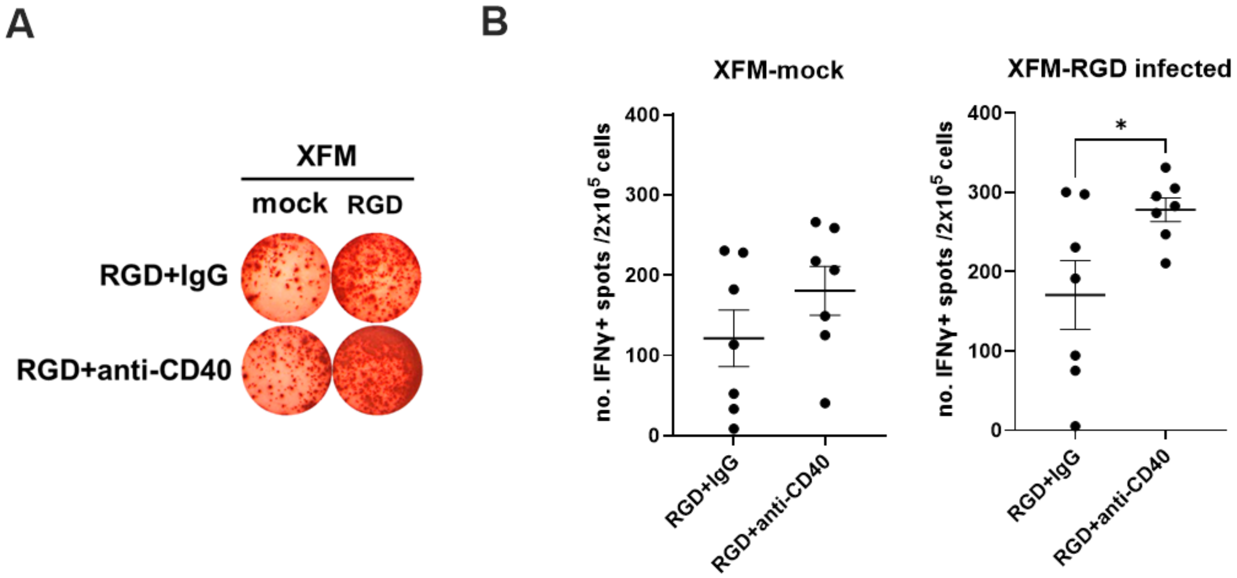

Figure S1: The anti-CD40 agonist enhances the Delta-24-RGD-specific adaptive immune response. Related to **Figure 2.**

(A) Representative picture of the wells from the ELISpot plate co-culturing RGD- or mock-infected XFM cells with splenocytes isolated from XFM-bearing mice at day six after being treated with the virus alone or the combination. (B) Analyses of IFNγ-producing splenocytes from the indicated groups measured by ELISpot 72h upon co-culturing with XFM cells. Paired T-test and Wilcoxon test were used for statistical analysis of the ELISpot. n =6-7 samples per group. ($p<0.05$*)

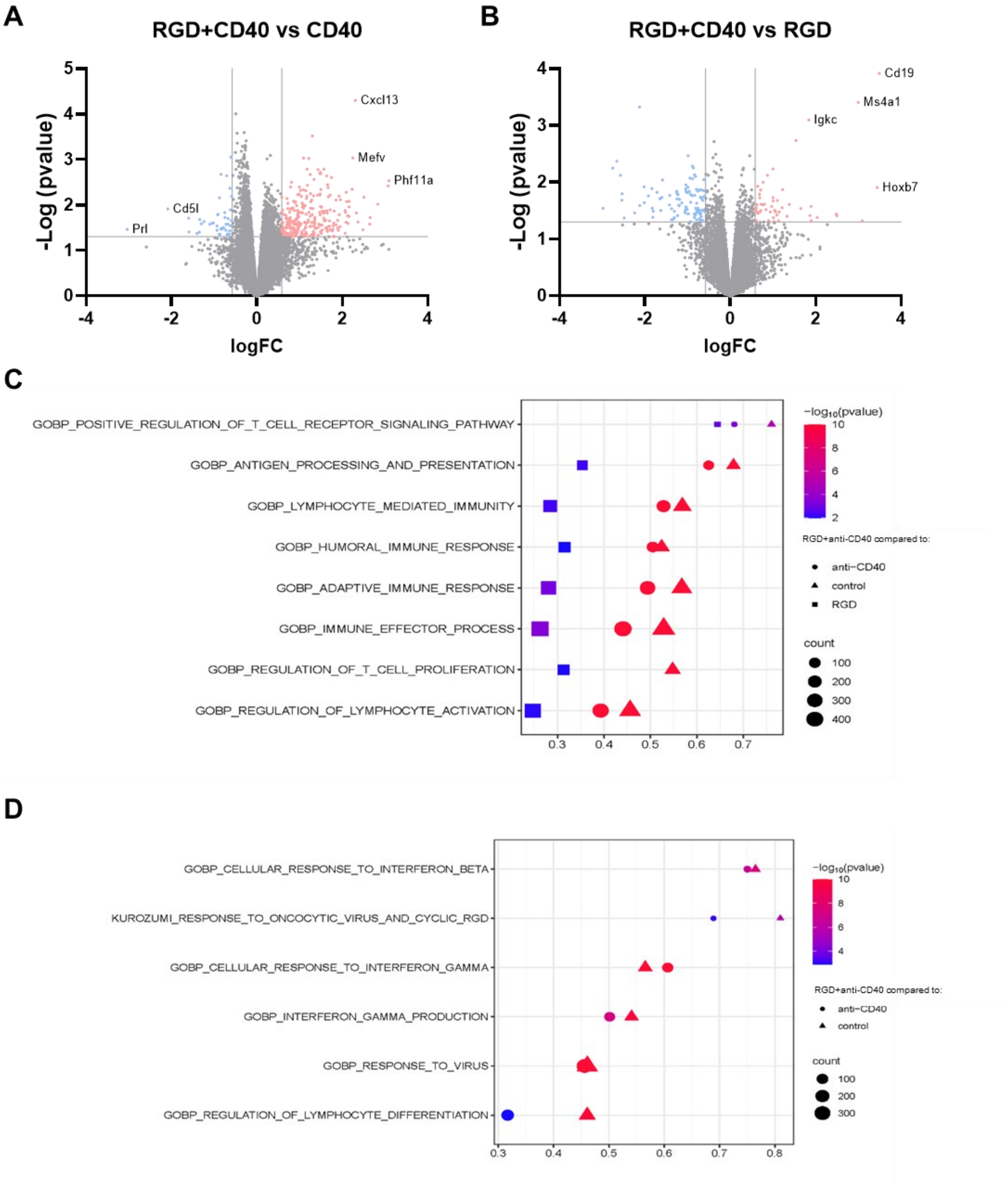

Figure S2. Transcriptional enrichment in immune-related genes and pathways that involve immune response in tumors treated with RGD+anti-CD40. Related to Figure 4.
(A-B) Volcano plots showing differentially expressed genes (DEG) from RNAseq of the bulk XFM tumors treated with the combination and compared to anti-CD40 or Delta-24-RGD alone at day 6. (C-D) Gene Set Enrichment Analyses within the combination and the indicated group of treatments, showing immune-related pathways expressed in the tumors at day 6 post-therapy. T-tests were used for statistical analyses. n =6 samples per group of treatment.

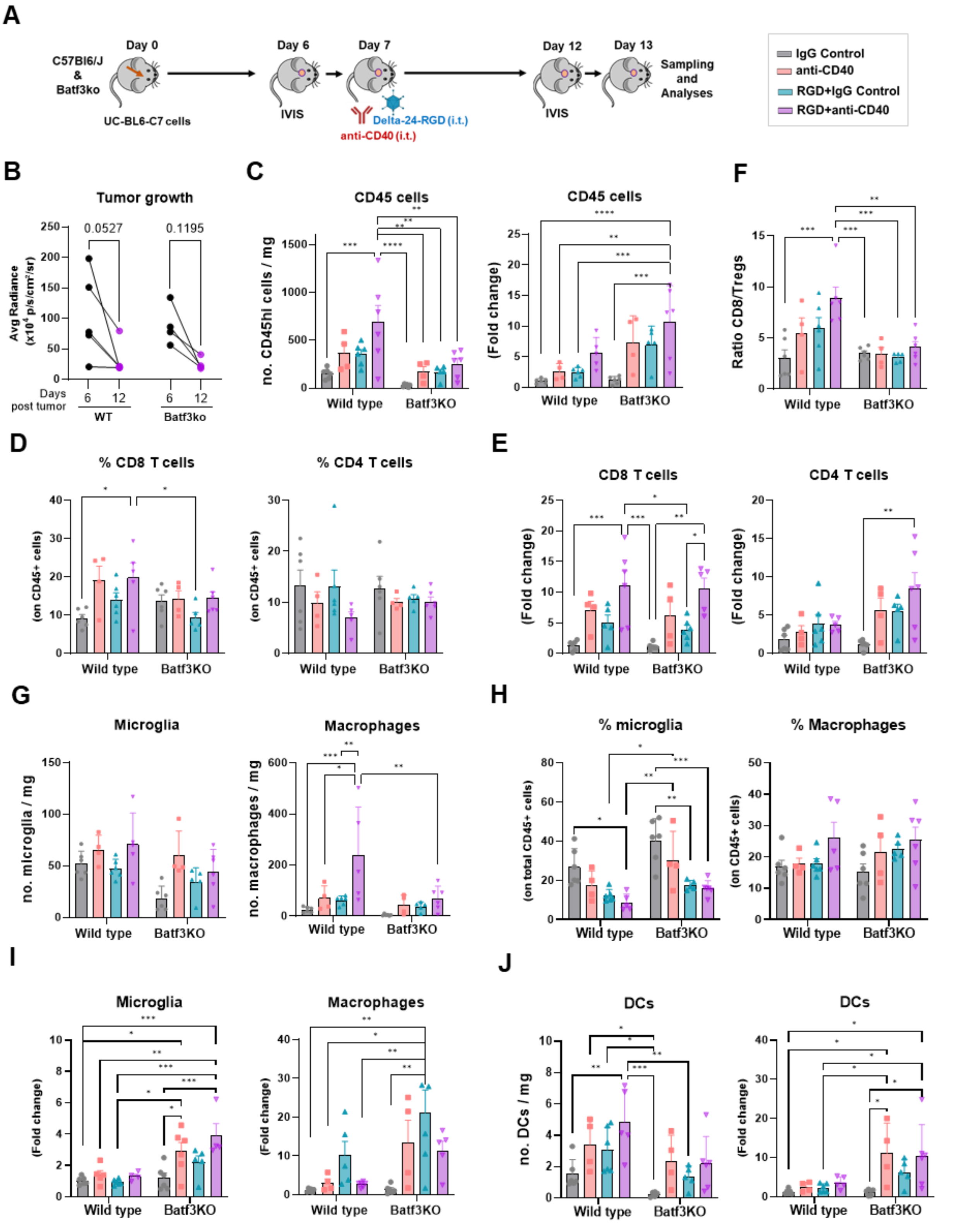

Figure S3. Tumor immune response UC-BL6-C7-bearing Batf3KO mice after the combination therapy. Related to Figure 4.

(A) Schedule of the experimental procedures performed in UC-BL6-C7-bearing C57Bl6/J and Batf3KO mice to study the immune response by flow cytometry. (B) Tumor growth decrease five days after treatment with the combination in wild type and Batf3KO mice. (C) Numbers of the immune infiltrating cells (CD45 high expressing cells) per tumor mg, and their fold change related to the mean of the respective IgG control groups. (D) Frequencies of CD8 and CD4 T

lymphocytes per mg of tumor. (E) Fold-change of CD8 and CD4 T cells in the indicated treatment related to the mean of the respective IgG control groups. (F) CD8/Tregs ratio measured in the tumor. (G-I) Numbers of microglia (CD45loCD11b+) and macrophages (CD45hiCD11b+) per mg, their frequency, and fold-change from UC-BL6-C7-tumors. (J) Numbers of DCs per tumor mg and DCs fold-change at day six post-treatment. Two-way ANOVA test was used for statistical analyses. n =4-6 samples per group. (p<0.05*; p<0.01**; p<0.001***; p<0.0001***)

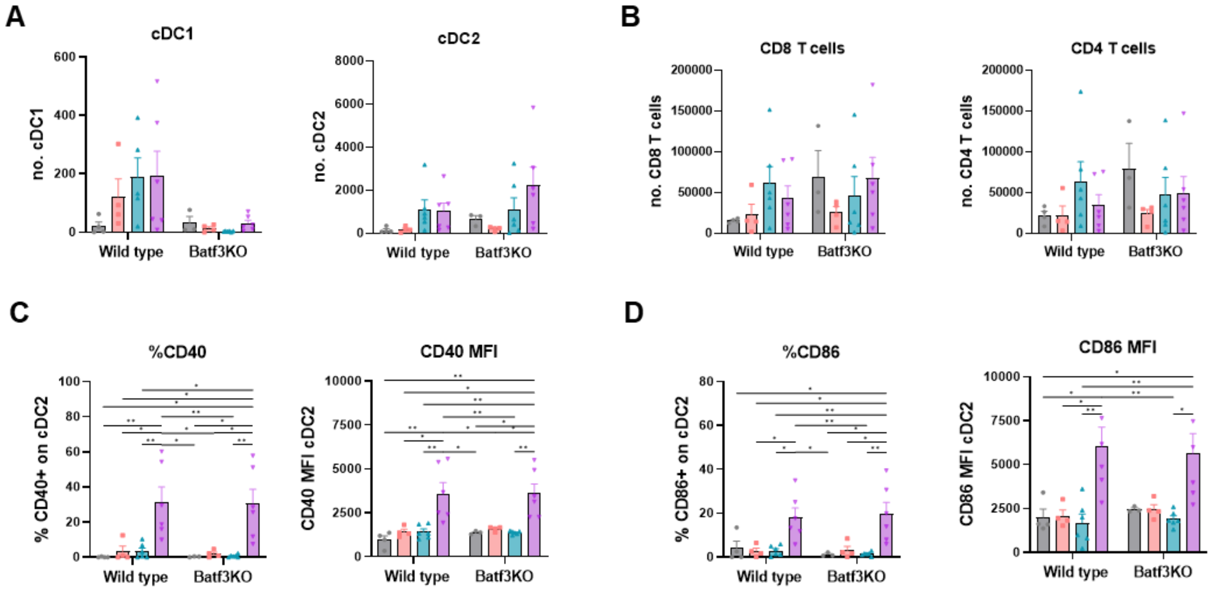

Figure S4. Batf3ko mice show similar cDC2 activation and T cell numbers in the draining-lymph node as wild type mice. **Related to Figure 4.**

(A and B) Absolute numbers of conventional DC (cDC1 and cDC2) and T lymphocytes (CD8 and CD4 T cells) obtained from deep cervical lymph nodes six days following the indicated treatments. (C and D) Frequency and expression (as Mean of Fluorescence Intensity, MFI) of CD40 and CD86 co-stimulatory receptors on cDC2 from draining lymph nodes in wild type and Batf3ko mice. Two-way ANOVA test was used for statistical analyses. n =4-6 samples per group. (p<0.05*; p<0.01**; p<0.001***; p<0.0001***)

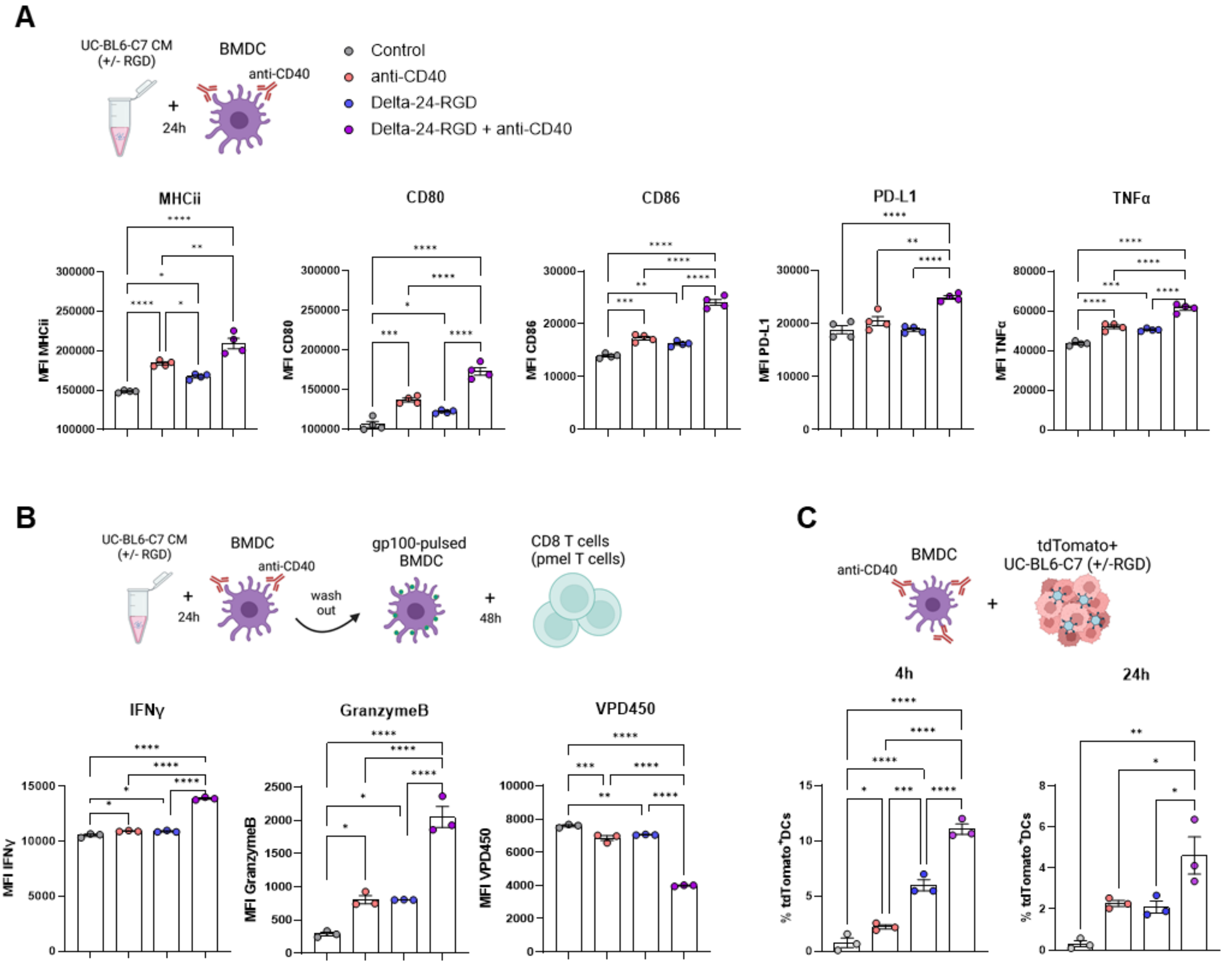

Figure S5. The Delta-24-RGD and the anti-CD40 agonist synergize in DC maturation, antigen presentation, and tumor phagocytic capability. Related to Figure 4.

(A) In vitro maturation assay of bone marrow-derived type 1 DCs (BMDC) incubated with the supernatant of RGD-transduced UC-BL6-C7 cells in the presence of IgG control or the anti-CD40 agonist antibody. Expression of MHC class II, CD80, CD86, and PDL1 maturation markers and the production of TNFα were assessed in cDC1 cells by flow cytometry 24 hours later. (B) Antigen presentation assay to pmel CD8 T cells with mature BMDC from A pulsed with the gp100 peptide. Graphs show the production of T cell activation (IFNγ) and cytotoxic markers (Granzyme B), and the proliferative capacity of CD8 T cells (decrease in VP450 cell dye) measured by flow cytometry 48 hours after. (C) Percentage of BMDC positive for tdTomato four and 24 hours after being cocultured with tdTomato+ UC-BL6-C7 transduced or not with the RGD virus. MFI: mean of fluorescence intensity. One-way ANOVA and the Kruskal-Wallis test were used for statistical analyses. n =4-6 samples per group. ($p<0.05$*; $p<0.01$**; $p<0.001$***)

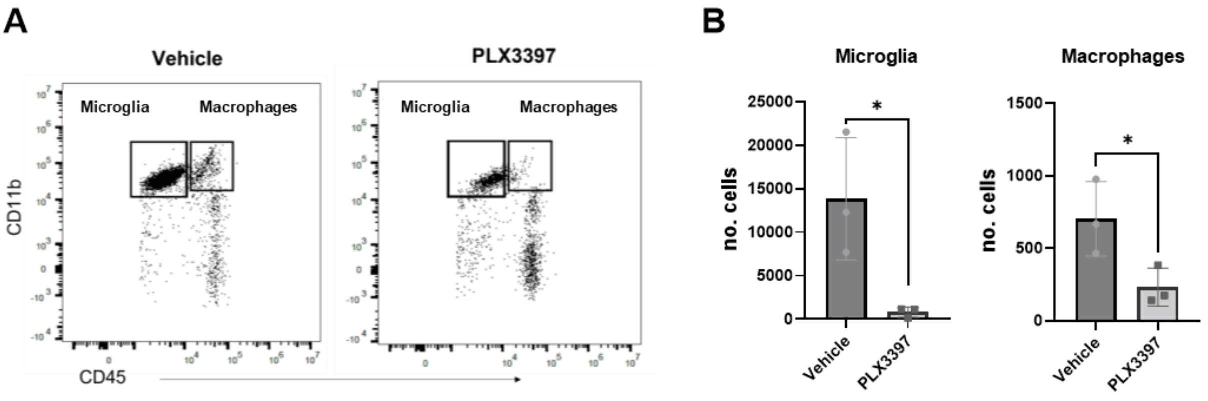

Figure S6. PLX3397 reduces the number of microglia and macrophages the tumor. Related to Figure 5.
(A) Representative example of microglia ($CD45^{lo}CD11b^{+}$) and macrophages ($CD45^{hi}CD11b^{+}$) from XFM-tumors three days upon starting the treatment with 50mg/kg of PLX3397 or the vehicle. (B) Quantification of the number of microglia and macrophages present in the tumors. The Mann–Whitney test was used for a number of cell analyses (n = 3 mice per group). ($p<0.05$*)

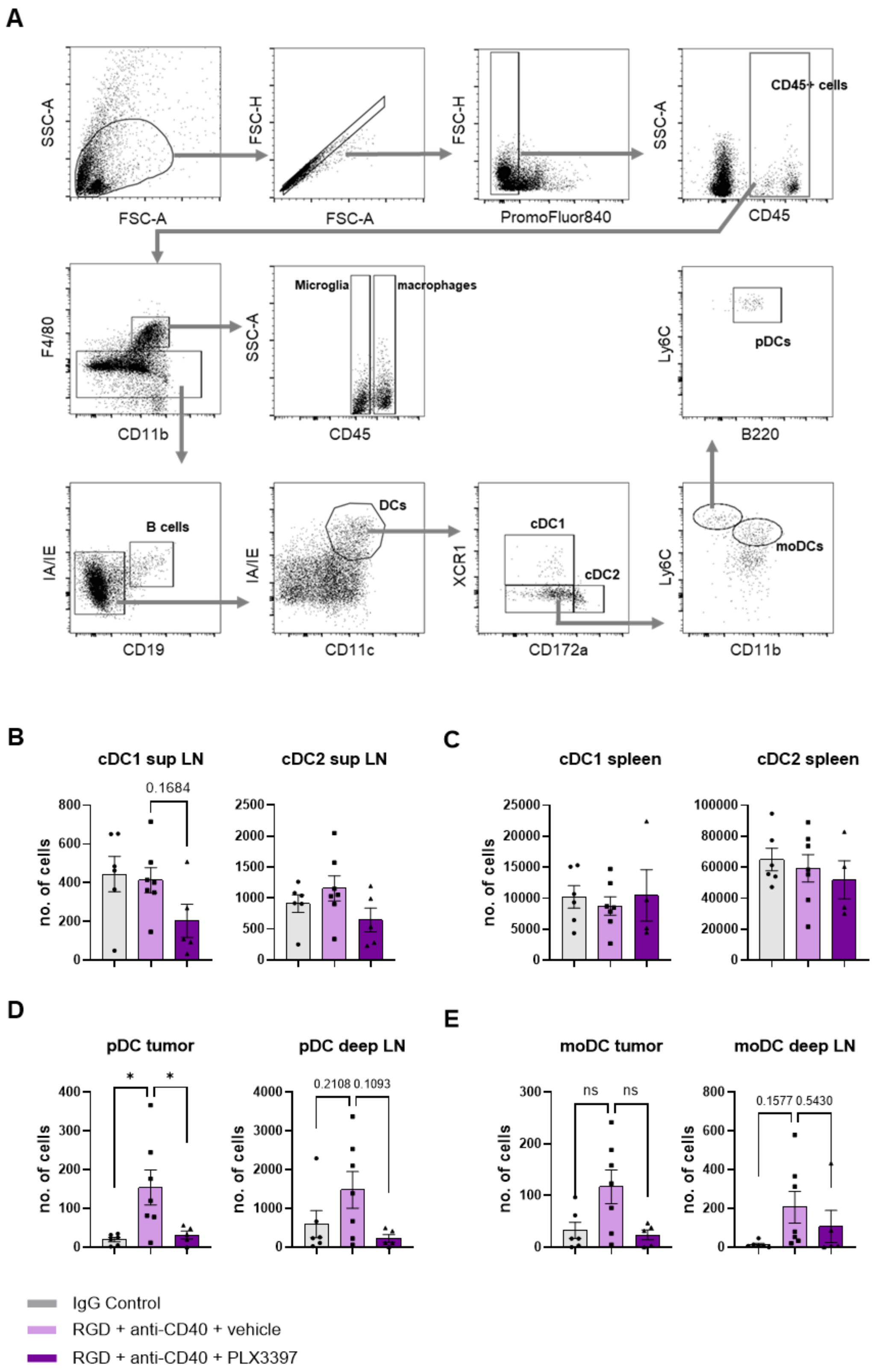

Figure S7. Effect of the inhibition of CSF1R in the recruitment of different types of DCs. Related to Figure 5. (A) Gating strategy followed to analyze the infiltration of the indicated DCs types in the tumor, lymph nodes and spleen. (B-C) Flow cytometry analyses of cDC1 and cDC2 infiltration of XFM-bearing mice in the superficial non-draining lymph nodes and the spleen six days post-treatment. (D-E) Numbers of monocytic DCs (moDCs) and plasmacitoid DCs (pDCs) in the tumor and deep draining lymph nodes 6 days post-treatment. One-way ANOVA and the Kruskal-Wallis test were used for statistical analyses from C-F data. n =6-7 samples per group. ($p<0.05$*)
