# Supplementary material for: Targeting the CD40 costimulatory receptor to improve virotherapy efficacy in diffuse midline gliomas
Source: Cell Rep Med. 2025 Jun 26;6(7):102204. doi: 10.1016/j.xcrm.2025.102204 (PMC12281365; doi:10.1016/j.xcrm.2025.102204)
Supplement: Document S2. Article plus supplemental information [file mmc2.pdf]

Cell Reports
Medicine

Article

Targeting the CD40 costimulatory receptor to improve virotherapy efficacy in diffuse midline gliomas

Graphical abstract

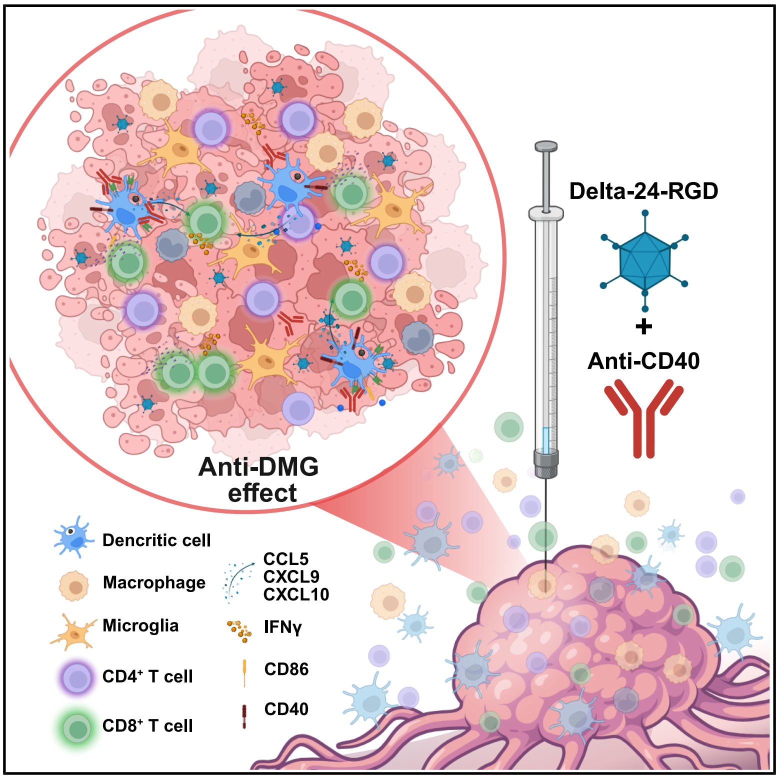

Authors

Sara Labiano, Javier Marco-Sanz,
Iker Ausejo-Mauleon, ...,
Jaime Gallego Perez-Larraya,
Ana Patiño-Garcia, Marta M. Alonso

Correspondence

slalminana@unav.es (S.L.),
mmalonso@unav.es (M.M.A.)

In brief

Labiano et al. show that co-administration of the Delta-24-RGD virus and a CD40 agonistic antibody is safe, yielding complete responses in DMG-bearing mice. The combination relies on type 1 conventional dendritic cells (cDC1s), which remodel the tumor microenvironment into a proinflammatory landscape, fostering long-term anti-tumor immunity against this challenging tumor.

Highlights

- CD40 agonism boosts Delta-24-RGD efficacy in pediatric DMG models
- cDC1s are essential for the long-lasting anti-tumor effect of Delta-24-RGD and anti-CD40
- Microglia and tumor macrophages contribute to the cDC1 recruitment following the therapy

Labiano et al., 2025, Cell Reports Medicine *6*, 102204
July 15, 2025 © 2025 The Author(s). Published by Elsevier Inc.
https://doi.org/10.1016/j.xcrm.2025.102204

CellPress

Cell Reports Medicine

CellPress
OPEN ACCESS

Article

Targeting the CD40 costimulatory receptor to improve virotherapy efficacy in diffuse midline gliomas

Sara Labiano,[1,2,3,*] Javier Marco-Sanz,[1,2,3] Iker Ausejo-Mauleon,[1,2,3] Virginia Laspidea,[1,2,3] Reyes Hernández-Osuna,[1,2,3] Marc Garcia-Moure,[1,2,3] Daniel de la Nava,[1,2,3] Sara Nuin,[1,2,3] Marisol Gonzalez-Huarriz,[1,2,3] Timothy N. Phoenix,[4] Ibon Tamayo,[5] Marta Zalacain,[1,2,3] Andrea Lacalle,[1,2,3] Lucía Marrodan,[1,2,3] Montserrat Puigdelloses,[1,2,3] Irati Hervás-Corpión,[1,2,3] Maria C. Ochoa,[1,2,3] Noelia Casares,[1,2,3] Oren J. Becher,[6] Candelaria Gomez-Manzano,[7] Juan Fueyo,[7] Jaime Gallego Perez-Larraya,[1,2,3] Ana Patiño-Garcia,[1,2,3] and Marta M. Alonso[1,2,3,8,*]

[1]Department of Pediatrics, Clinica Universidad de Navarra, Pamplona, Spain
[2]Program in Solid Tumors, Center for the Applied Medical Research (CIMA), University of Navarra, Pamplona, Spain
[3]Health Research Institute of Navarra (IdiSNA), Pamplona, Navarra, Spain
[4]Division of Pharmaceutical Sciences, James L. Winkle College of Pharmacy, University of Cincinnati, Cincinnati, OH, USA
[5]Bioinformatics Platform, Center for the Applied Medical Research (CIMA), University of Navarra, Pamplona, Spain
[6]Jack Martin Fund Division of Pediatric Hematology-oncology, Mount Sinai, New York, NY, USA
[7]Dpt. Of NeuroOncology, UT MD Anderson Cancer Center, Houston, TX, USA
[8]Lead contact
*Correspondence: slalminana@unav.es (S.L.), mmalonso@unav.es (M.M.A.)
https://doi.org/10.1016/j.xcrm.2025.102204

SUMMARY

Diffuse midline glioma (DMG) is a devastating pediatric brain tumor. The oncolytic adenovirus Delta-24-RGD has shown promising efficacy and safety in DMG patients but is not yet curative. Thus, we hypothesized that activating dendritic cells (DCs) through the CD40 costimulatory receptor could increase antigen presentation and enhance the anti-tumor effect of the virus, resulting in long-term responses. This study shows that the intratumoral co-administration of Delta-24-RGD and a CD40 agonistic antibody is well tolerated and induces long-term anti-tumor immunity, including complete responses (up to 40%) in DMG preclinical models. Mechanistic studies revealed that this therapy increased tumor-proliferating T lymphocytes and proinflammatory myeloid cells, including mature DCs with superior tumor antigen uptake capacity. Moreover, the lack of cross-presenting DCs and the prevention of DC recruitment into the tumor abolish the Delta-24-RGD+anti-CD40 anti-DMG effect. This approach shows potential for combining virotherapy with activating antigen-presenting cells in these challenging tumors.

INTRODUCTION

Diffuse midline gliomas (DMGs) are aggressive glial tumors and the leading cause of death by a pediatric tumor.[1] The main peak incidence occurs in 5- to 9-year-old children, accounting for 10%–20% of primary brain tumors in this age group. Another small peak around 18 years old has been described, appearing also sporadically in some adult cases.[1,2] Most DMGs occur in the brainstem (also known as diffuse intrinsic pontine glioma, DIPG) but can also be found in other midline areas such as the thalamus and spinal cord.[3] Importantly, their location makes surgery an impossible therapeutic option in most cases. Currently, patients with DMG have very poor outcomes, with a survival of 9–12 months after the diagnosis.[4] The standard of care with radiotherapy is only palliative, and patients rapidly succumb to the disease, highlighting the urgent need for alternative therapeutic approaches to address this deadly disease.[5]

The development of immunotherapy for pediatric brain tumors is in the early stages, and, up to now, there are no immune-oncology therapies approved for them at diagnosis or relapse.[6] DMG represents a challenge for immuno-oncology because of the non-inflammatory tumor immune microenvironment, characterized by the lack of infiltrating lymphocytes and the abundance of myeloid cells.[7,8] Nevertheless, the feasibility of several immunotherapeutic approaches, including immunomodulatory antibodies, chimeric antigen receptor (CAR)-T cells, or oncolytic viruses, is currently being demonstrated in these patients.[9] Still, the impact on the survival of therapies targeting T cells, such as immune checkpoint inhibitors, remains to be proven. The absence of tumor-infiltrating T lymphocytes (TILs) could explain the poor results obtained targeting PD1 and CTLA-4 in the clinic.[10,11] Successful preclinical data with CAR-T cell therapy position it as a promising approach for DMG patients.[12] Indeed, recent clinical trials have assessed various delivery routes and targets, including GD2 and B7H3, resulting in clinical and radiological benefits, but they are insufficient to be curative[13–15] and not exempt from severe secondary effects such as neuroinflammation.[14]

© 2025 The Author(s). Published by Elsevier Inc.
This is an open access article under the CC BY license (http://creativecommons.org/licenses/by/4.0/).

Due to scarce therapeutic options in difficult-to-treat tumors such as DMGs, oncolytic viruses are another biological agent being evaluated in this scenario. In this sense, the oncolytic virus Delta-24-RGD represents a non-toxic alternative strategy for treating DMG patients. This replication-competent adenovirus was designed to replicate in and destroy tumor cells with an aberrant retinoblastoma (RB) pathway.[16,17] We have previously demonstrated that the local inoculation of the Delta-24-RGD (DNX-2401 in the clinic) is safe and increases the overall survival up to 17.8 months in newly diagnosed pontine DMG patients.[18,19] In addition, Delta-24-RGD can modulate the tumor immune microenvironment, favoring the recruitment of T cells.[18,19] However, such a proinflammatory scenario is insufficient to be curative.

In solid tumors, including brain cancer, successful and durable anti-tumor T cell responses mainly depend on effective priming by antigen-presenting cells (APCs) and the subsequent activation status in the tumor microenvironment.[20] Maturation and antigen presentation capabilities of APCs, such as B cells, dendritic cells (DCs), and macrophages, are increased upon ligation of the costimulatory receptor CD40 to its natural ligand (CD40L) that is present on activated T cells.[21] Indeed, the CD40 signaling in DCs produces a more efficient priming of T lymphocytes resulting in a robust immunological memory generation.[22] The activation of CD40 favors a proinflammatory phenotype in tumor-associated macrophages, inducing their killing capabilities and supporting anti-tumor T cell responses.[23] In microglia, the resident macrophages of the brain, the CD40 receptor plays an important role in their activation and the production of proinflammatory cytokines in the context of neuroinflammatory diseases.[24] In addition, the targeting of CD40 with agonistic monoclonal antibodies (mAb) has shown effective results in different cancer types, such as pancreatic cancer, where both the adaptive and innate immune systems drive the main mechanisms of action of the CD40-targeted therapy.[22,25] In an adult glioma preclinical model, using a CD40 mAb enhances the anti-tumor efficacy of tumor lysate-based vaccines.[26] However, given as a single agent, the intracranial administration of this antibody results in less efficiency in the same preclinical setting, suggesting the need to combine this route of administration with other therapies.[26] Therefore, we decided to combine the oncolytic and immunomodulating effect of the Delta-24-RGD with targeting APCs through the CD40 receptor to treat DMGs.

Here, we show that targeting CD40 with a local injection of an agonistic antibody is safe and enhances the anti-tumor effect of the Delta-24-RGD in DMG orthotopic mouse models. The combination generates long-term responses that rely on the adaptive immune system and the presence of cross-presenting DCs. Moreover, other APCs present in the brain, such as microglia and tumor-associated macrophages, play a crucial role in the recruitment of DCs into tumors and, hence, in the survival benefit of the therapy. These results show the translational potential of local combinations of virotherapy with the targeting of DCs through the CD40 activation in a dismal disease such as DMG.

RESULTS

The combination of Delta-24-RGD with a CD40 agonistic antibody increases the survival of DMG-bearing mice

First, to buttress our hypothesis, we evaluated the efficacy of the Delta-24-RGD+CD40 agonist antibody combination in an H3 wild-type DMG model (XFM cell line; schedule from Figure 1A). The administration of Delta-24-RGD or the anti-CD40 agonist as single treatments did not lead to a survival advantage in this model (14 and 16 days, respectively). However, the intratumoral co-treatment of both agents significantly extended the survival of mice (median survival of 24.5 days), leading to 40% of complete responses (Figure 1B). Timing and sequence are critical for the success of immunotherapeutic combinatorial strategies,[27] and our previous data with an armed Delta-24-RGD virus showed changes in the tumor immune response ranging from 7 to 10 days after treatment, specifically in the T cell infiltration.[28] Thus, we hypothesized that delaying the anti-CD40 administration could foster the antigen presentation to the recruited lymphocytes and result in a superior therapeutic effect. To address this point, we treated DMG-bearing mice with the Delta-24-RGD and injected the CD40 agonist 3 days later (Figure 1C). The sequential schedule showed no differences in survival compared to those mice treated with the immunoglobulin G (IgG) control (median survival of 13 days; Figure 1D). Again, the intratumoral co-treatment of both agents significantly extended the survival of mice (median survival of 24 days) and resulted in 37.5% of long-term survivors (3/8) (Figure 1D). Next, we evaluated the tumor kinetics upon the co-treatment schedule using luminescence in a subset of these mice. We showed that the tumor growth was also delayed in the combination group, and the rate significantly decreased (3.65 times less) compared to that of IgG control-treated mice (Figures 1E–1G).

Likewise, the anti-tumor efficacy of the combination was corroborated in mice bearing a different syngeneic cell line, the UC-BL6-C7, that harbors a DMG driver mutation in the Histone 3 gene (H3K27M).[29] Unlike both monotherapies (with a median survival of 60 days), the Delta-24-RGD and the anti-CD40 simultaneously injected prolonged the overall survival of treated mice (64 days) compared to IgG control-treated animals (48 days) (Figure 1H). Finally, none of the treated mice showed signs of toxicity. Indeed, the lack of body weight loss observed in both DMG models early upon treatment indicated that the combination was well tolerated (Figure 1I).

These results indicate that, to have an effect, the CD40 signaling must be locally activated early on while the Delta-24-RGD virus exerts its action promoting inflammation in the tumor context.

The Delta-24-RGD and anti-CD40 combination confers a long-lasting protective immunological memory

We previously reported that, in mouse syngeneic models, the anti-tumor effect of the virus is dependent mainly on the local inflammation generated upon infection rather than on its oncolytic action due to the absence of replication in mouse cells.[19] In line with this, the Delta-24-RGD+anti-CD40 treatment completely lost efficacy in Rag2Il2r$\gamma^{-/-}$ immunodeficient mice compared to IgG control in both the XFM and the UC-BL6-C7 models (Figures 2A and 2B). In addition, long-term responder mice (that rejected the

CellPress
OPEN ACCESS

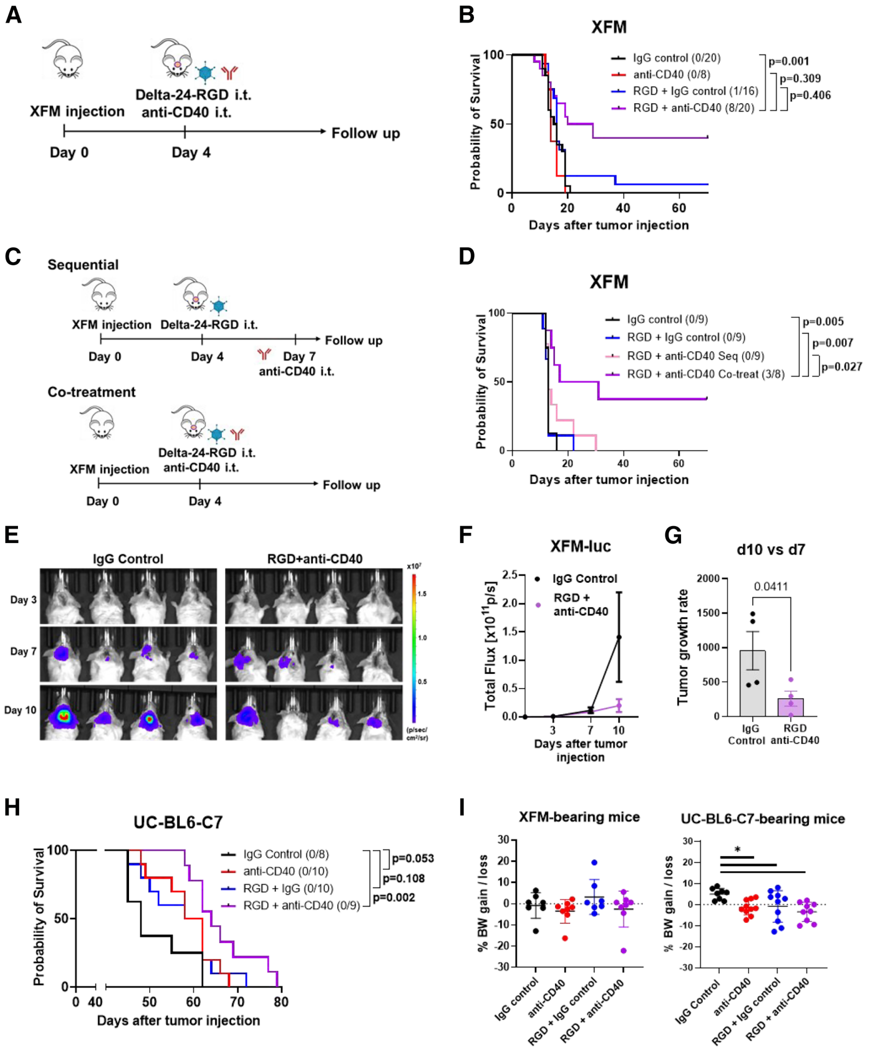

Figure 1. The combination of Delta-24-RGD with the anti-CD40 is safe and produces complete responses in DMG-bearing mice

(A) Experimental schedule combining the treatment with anti-CD40 (24 μg; clone FGK4.5 intratumoral [i.t.]) and the oncolytic virus Delta-24-RGD (10^7 plaque-forming unit [PFU], i.t.) in the XFM tumor model.

(B) Survival of XFM-bearing mice treated 4 days after tumor injection.

(C) Experimental schedule combining the treatment with anti-CD40 (24 μg; clone FGK4.5 i.t.) the same day or 3 days after virus injection (10^7 PFU of Delta-24-RGD i.t.).

(D) Survival curves of XFM-bearing mice treated with the indicated schedules.

(E) Images of bioluminescence were obtained from XFM-bearing mice treated with IgG control or the combination at the indicated time points.

(F) Measurement of tumor growth over time by bioluminescence.

(G) Tumor growth change at day 10 compared to day 7 post-treatment in mice from (E).

(H) Survival of UC-BL6-C7-bearing mice treated 7 days after tumor injection.

(I) Percentage of body weight change of DMG-bearing mice on day 3 compared to the day before mice were treated with the indicated agents.

BW, body weight. Error bars represent mean ± SEM. The log rank test was used for statistical analysis comparing the indicated groups in the survival experiments. n = 8–20 mice per group. One-way ANOVA and the corresponding non-parametric test were used to statistically analyze body weight. n = 7–10 mice per group. The Mann-Whitney test was used for tumor growth rate analysis (n = 4 mice per group) *$p < 0.05$.

XFM tumor; Figure 1B) controlled the tumor growth of a rechallenge performed with the same cells, indicating the generation of long-term anti-tumor immune memory (Figure 2C). These data confirmed that the effect of the combination is still due to the adaptive immune system. Interestingly, 6 days after treatment, the combination showed higher numbers of interferon (IFN)γ-producing lymphocytes than the virus alone when splenocytes from both groups of mice were *ex vivo* restimulated with Delta-24-RGD-infected XFM cells (Figure S1), indicating that the peripheral immune response was mainly directed to viral antigens at early time points. However, in the presence of FTY720, a drug that avoids the egress of lymphocytes from lymph nodes to the periphery, long-term responder mice were able to reject a rechallenge, suggesting the formation of local and resident immune memory against tumor cells (Figure 2D).

The combination therapy remodels the tumor immune microenvironment

The previous observations prompted us to interrogate the immune context at the tumor site after the combination. Thus, we characterized the tumor microenvironment 6 days after treatment by flow cytometry (Figure 3A). The combination of Delta-24-RGD and anti-CD40 leads to increased CD45 immune cells, CD8 and CD4 T lymphocytes, and regulatory T cells (Tregs) compared to IgG control- and anti-CD40-treated groups (Figures 3B–3E). The virus alone also increased such populations. However, its effect on the proliferative tumor-infiltrating lymphocytes (TILs) was significantly lower than the combination (Figure 3F). In addition, $CD4^+$ and $CD8^+$ TILs from the combination group showed a more active phenotype, with higher levels of molecules such as CD137, TIM3, and PD1 (Figures 3G and 3H). Other lymphoid components, such as natural killer (NK) cells, did not change upon treatment (Figure 3I).

The study of the tumor myeloid compartment showed, that while the number of microglia increased with the anti-CD40, the levels of macrophages and monocytes were significantly higher with the virus and the combination compared to IgG control- or anti-CD40-treated groups (Figure 4A). Similarly, DC numbers were augmented in both groups containing the virus (Figures 4B–4D). However, their frequency was significantly higher upon the combination compared to the rest of the groups, including Delta-24-RGD alone (Figure 4E). Proinflammatory

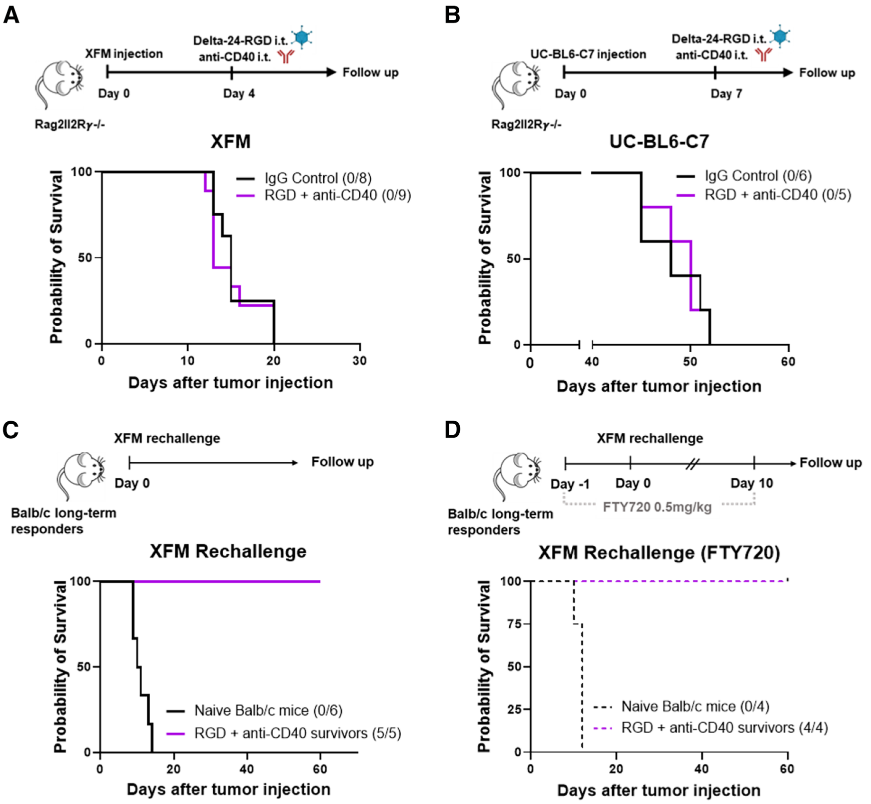

Figure 2. Delta-24-RGD and anti-CD40 therapy promotes a durable anti-tumor immune response

(A and B) Survival curves of Rag2Il2R$\gamma^{-/-}$ immunodeficient mice bearing XFM and UC-BL6-C7 tumors; treated as indicated in the experimental schedule.

(C) Survival curves of long-term survivors subjected to rechallenge with XFM cells as shown in the experimental illustration.

(D) Survival curves of Delta-24-RGD+anti-CD40-long-term survivors subjected to FTY720 and rechallenged with XFM cells in the pons.

Log rank test was used for statistical analysis comparing the indicated group with untreated mice in the survival experiments. $n = 4–9$ mice per group.

chemokines typically produced by DCs to promote the recruitment of T cells, such as CCL5, CXCL9, and CXCL10, were increased in the tumor microenvironment of mice treated with the combination (Figure 4F). We also observed higher levels of IFNγ, which aligns with the presence of proliferating TILs (Figure 4F). Transcriptomic analysis of the tumor bulk showed 957 differentially expressed genes (DEGs; 548 upregulated and 409 downregulated) within the combination and the IgG control group (Figure 4G). Some of them are immune-related genes typically expressed by B cells (*Ms4a1* and *Cd19*), macrophages and microglia (*Cd68*), and DCs (*Clec9a*). Others correspond to immune cell activation (*Cd80* and *Cd69*) and proinflammatory chemokines (*Ccl5*, *Ccl22*, and *Cxcl13*) (Figure 4G). Compared to the anti-CD40 and the Delta-24-RGD alone, the combination showed 623 DEGs (412 upregulated and 211 downregulated) and 148 DEGs (49 upregulated and 99 downregulated), respectively (Figures S2A and S2B). In addition, tumors treated with the Delta-24-RGD and anti-CD40 were enriched in gene signatures involved in antigen processing and presentation, lymphocyte-mediated immunity, and regulation of T lymphocyte activation, among others (Figure S2C). The combined treatment showed an increase in genes related to the Delta-24-RGD virus response, as well as to IFNs (IFNβ and IFNɣ) (Figure S2D). Additionally, there was an increase in pathways related to macrophage activation, DC differentiation, and CD40 signaling in the tumors treated with the combination, compared to those treated with IgG control or anti-CD40 alone (Figure 4H).

Collectively, these data showed that the combination remodels the tumor immune microenvironment, not only the lymphocytes but also the myeloid cells, toward a proinflammatory scenario.

DCs play an essential role in the anti-tumor immune response after Delta-24-RGD and anti-CD40 therapy

In line with RNA sequencing (RNA-seq) data, microglia and tumor-associated macrophages exhibited higher levels of major histocompatibility complex (MHC)-II molecules than the other groups (Figure 4I). This indicates the development of a proinflammatory phenotype in the tumor niche after the combination. Moreover, there was an increase in the number of DCs expressing high-intensity MHC-II on their membrane (Figure 4J), as well as costimulatory receptors CD40 and CD86 (Figure 4K), showing a more mature phenotype upon the combination. In line with this, there is a tendency for PDL1 expression to decrease after the combination compared to Delta-24-RGD (Figure 4L), which is associated with an enhanced anti-tumor T cell response.[30]

Using a DMG model expressing the mCherry fluorescent protein, we explored the antigen uptake capacity of the tumor myeloid compartment (focusing on DCs, macrophages, and microglia). The proportion of phagocytic tumor DCs, which are positive for the mCherry protein, is significantly higher in the combination treatment compared to the control. Interestingly, the combination did not improve tumor cell phagocytosis in tumor macrophages and microglia (Figures 4M and 4N). Indeed, the percentage of mCherry+ tumor macrophages increased with the anti-CD40 alone as compared to the Delta-24-RGD virus and the combination (Figures 4M and 4N). The administration of Delta-24-RGD and anti-CD40 was able to initially control tumor growth in *Batf3ko* mice lacking cross-presenting DCs (type 1 conventional dendritic cell [cDC1]), similar to the wild-type mice (Figures S3A and S3B). However, it did not provide any survival benefit, and tumors from both IgG control- and combination-treated *Batf3ko* mice were similar in size at the

CellPress
OPEN ACCESS

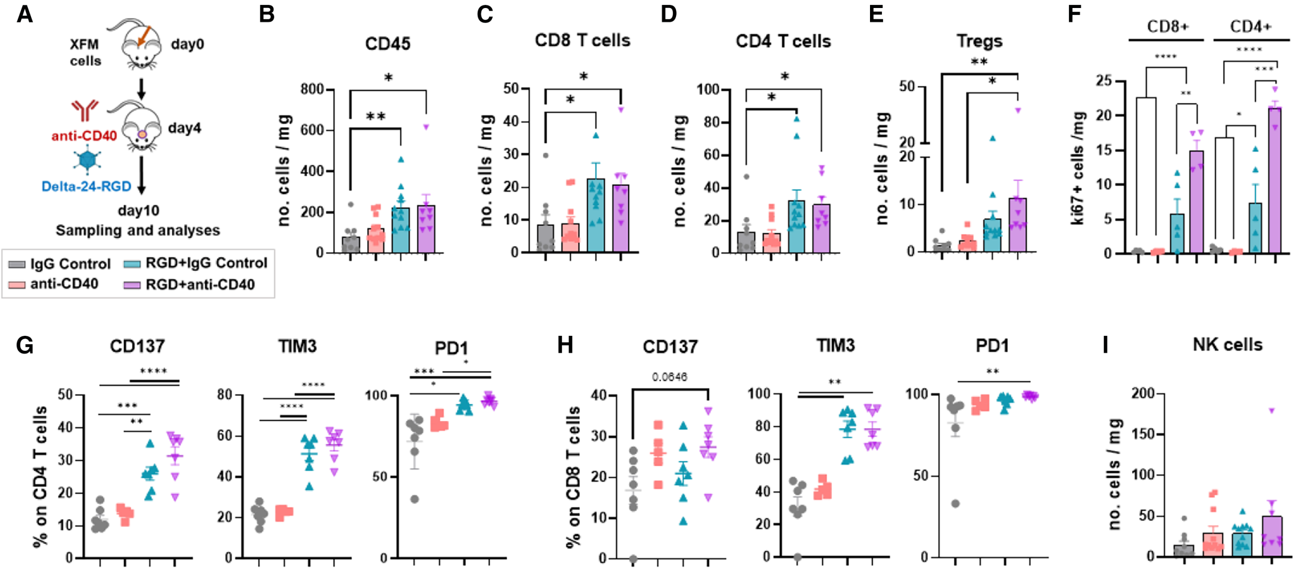

Figure 3. The combination increases proliferating tumor-infiltrating lymphocytes

(A) Schedule of the experimental procedures performed in the tumor microenvironment study in XFM tumors 6 days after the indicated treatments.

(B–E) Numbers of the total immune-infiltrating populations (CD45 high expressing cells), CD8 and CD4 T lymphocytes, and regulatory T cells (Tregs) per mg of tumor assessed by flow cytometry.

(F) Number of proliferating CD8 and CD4 T cells (measured by ki67-expressing cells) upon the indicated conditions.

(G and H) Percentage of CD4 and CD8 T cells in the tumor expressing the indicated activation molecules.

(I) Numbers of natural killer (NK) cells per tumor mg at day 6 post-treatment.

Error bars represent mean ± SEM. One-way ANOVA and the Kruskal-Wallis test were used for statistical analyses. n = 5–10 samples per group. (*$p < 0.05$; **$p < 0.01$; ***$p < 0.001$; ****$p < 0.0001$).

endpoint (Figures 4O and 4P). The analysis of tumor immune components from *Batf3ko* mice revealed that, although there is less immune infiltrate compared to wild-type counterparts, the absence of cDC1 does not prevent the recruitment of immune cells (CD45hi-positive cells) following the combination (Figure S3C). The frequency of T cells among total CD45hi-positive cells remained similar in the knockout mice across the treatments. However, the combination increased the CD8 and CD4 T cells (Figures S3D and S3E), and the CD8/Tregs ratio was only increased with the combination in the wild-type mice (Figure S3F). Regarding myeloid cells, microglia and macrophages exhibited the same trend in wild-type and Batf3ko mice after treatments (Figures S3G–S3I). In contrast, the number of DCs did not change with the combination in the knockout mice, as observed in their wild-type counterparts (Figure S3J). Apart from the lack of cDC1 cells, the numbers of cDC2 and T cells (Figures S4A and S4B) and the maturation profile of cDC2 (Figures S4C and S4D) were similar in tumor-draining lymph nodes from both strains treated with the combination. *Ex vivo* maturation of cDC1 reveals that the anti-CD40 and Delta-24-RGD virus have a synergistic effect on the expression of MHC-II (IA/IE) molecules, maturation receptors, and proinflammatory cytokines (Figure S5A). Such cDC1 cells also exert a higher cross-presentation capacity, measured by IFNγ and Granzyme B production by CD8 T cells and their proliferation (Figure S5B). Tumor phagocytosis by cDC1 cells was also improved when CD40 signaling was activated, and tumor cells were previously infected with the Delta-24-RGD virus compared to single agents (Figure S5C). Overall, this confirms the significance of conventional DCs, particularly cDC1, in the anti-tumor effect of the combination therapy. Additionally, it suggests that, despite exhibiting a proinflammatory phenotype by upregulating MHC-II molecules, macrophages and microglia may not play as crucial a role in antigen presentation as DCs do.

CSF1R inhibition abrogates the survival benefit and tumor DC recruitment

The proinflammatory phenotype observed in microglia and macrophages with the upregulation of antigen presentation molecules encouraged us to study their role in the anti-tumor effect observed in the Delta-24-RGD+anti-CD40 combination. To this end, we selected the PLX3397 drug (also known as pexidartinib), which is widely used for the blockade of microglia and tumor-associated macrophages through the inhibition of the colony stimulating factor 1 receptor (CSF1R; Figures S6A and S6B). The treatment with PLX3397 resulted in the loss of the survival benefit of the combination therapy compared to the vehicle (Figures 5A and 5B). When examining the tumor microenvironment, PLX3397 abrogated the Delta-24-RGD+anti-CD40-dependent recruitment of DCs (Figure 5C). Additionally, there was a reduction in the number of DCs from the deep cervical draining lymph nodes (deep LNs) that did not occur in other lymphoid organs, such as the non-draining superficial cervical lymph nodes (sup LNs) or the spleen (Figure 5C). An exhaustive analysis of the DC subtypes (Figure S7A) showed that conventional DCs (cDC1 and cDC2) were the populations increased in the tumor and deep LNs

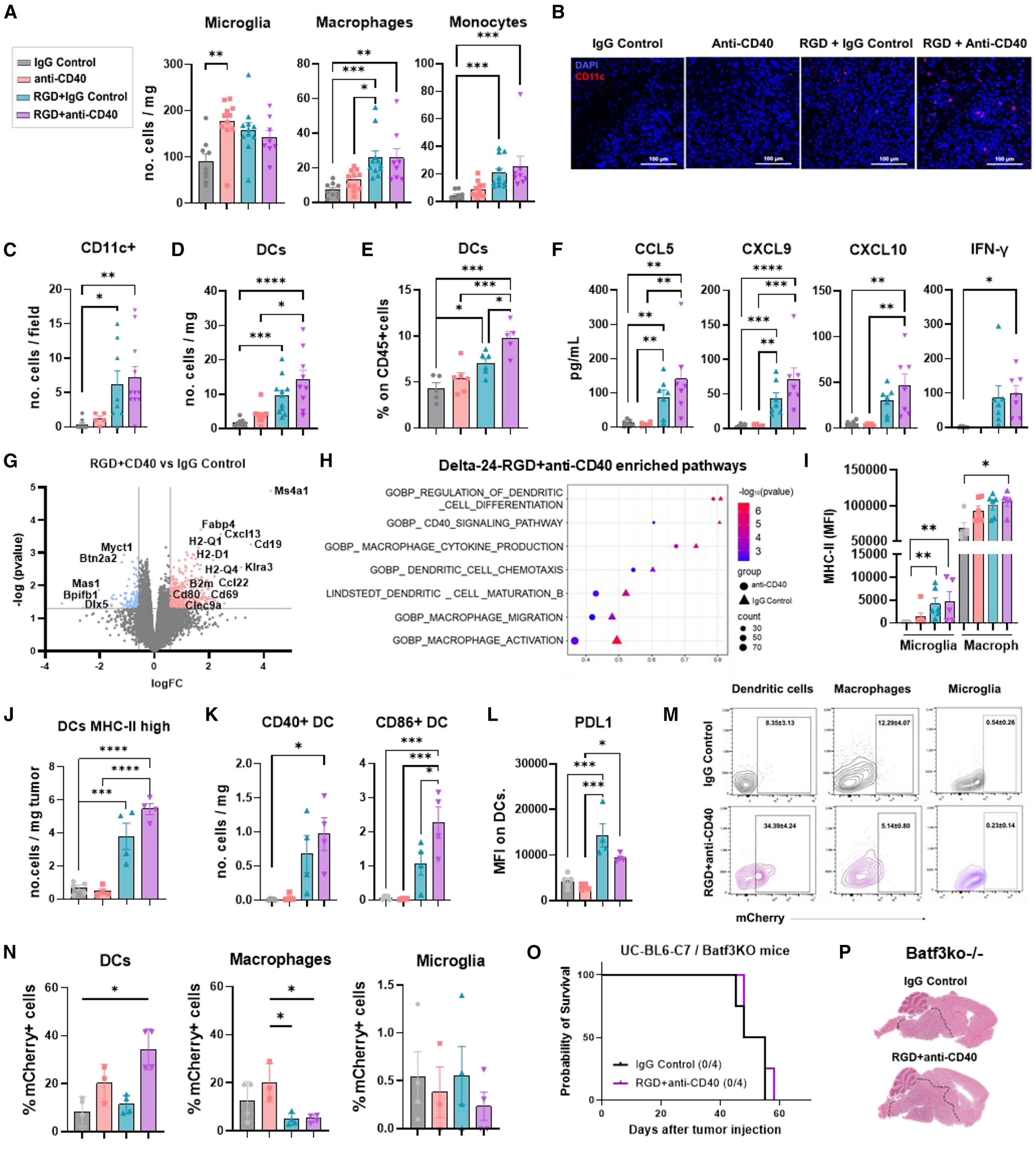

Figure 4. The combination enhances a proinflammatory phenotype of the tumor myeloid compartment, with cDC1 playing a pivotal role in the anti-tumor response

(A) Numbers of microglia, macrophages, and monocytes present per mg of tumor and assessed by flow cytometry in XFM tumors 6 days after being treated with the indicated conditions.

(B and C) Representative images and analysis of the number of CD11c-positive cells (dendritic cells) assessed by immunofluorescence staining of XFM tumors at day six post-treatment. Scale bar: 100 μm.

(D and E) Number and percentage of dendritic cells (DCs) from the total immune tumor infiltrate.

(legend continued on next page)

upon the combination, remaining unchanged in the sup LNs and the spleen (Figures 5D, 5E, S7B, and S7C). The numbers of tumor and deep LN plasmacytoid DCs (pDCs) also dropped with PLX3397 (Figure S7D). Finally, levels of monocytic DCs, although not significantly, were lower with PLX3397 than with the vehicle following the combination in both the tumor and the deep LN (Figure S7E). The inhibition of CSF1R also resulted in low tumor proinflammatory chemokines and cytokine abundance (Figure 5F). Altogether, these data emphasize the importance of tumor macrophages and microglia in recruiting DCs into the tumor and subsequently to the draining lymph nodes.

DISCUSSION

The safety and efficacy observed with the oncolytic virus Delta-24-RGD in DMG patients make it a promising therapy for this orphan disease for which current therapies have been inefficient so far.[31] However, there is still room to enhance the clinical benefit of this virus, and understanding the tumor immune microenvironment is crucial to achieve this. DMGs are characterized by a lack of T cell infiltration and an inert immune microenvironment mainly composed of myeloid cells.[7,32] Consequently, classical immunotherapeutic approaches focused on T lymphocytes have proven ineffective in these patients.[33] The field of immunotherapy is now shifting toward strategies that alter the composition and function of the tumor myeloid cells.[34] DCs, macrophages, and microglia are likely the first immune components infiltrating the tumor site. Thus, we decided to target these cells through the CD40 receptor to enhance the effectiveness of Delta-24-RGD by improving tumor phagocytosis and reversing dysfunctional antigen presentation to enhance T cell anti-tumor responses.

To combine both approaches, we initially considered generating an oncolytic virus expressing the CD40 ligand as other groups have done.[35–38] However, from our previous experience with armed Delta-24-RGD, we know it takes about 2–3 days for the costimulatory ligands to be expressed on infected cells *in vivo*.[39] Therefore, considering that the anti-CD40 agonist only shows effectiveness when co-injected with the virus and not 3 days later, we proceeded with the study of the Delta-24-RGD combined with the CD40 mAb. In addition, an oncolytic virus expressing CD40L showed an anti-tumor effect in brainstem immunocompetent models but was accompanied by neuroinflammation-associated toxicity.[40] By contrast, we have not observed such toxicity in our studies, which could be attributed to the use of different mouse models in the research. They used adult glioma cell lines that produce a tumor mass and are much more immune infiltrated than human DMG tumors. This high immune infiltration, combined with therapies that induce inflammation, may lead to neurotoxicity. In contrast, we have unique orthotopic mouse models of pediatric DMG that resemble genetically and phenotypically the human disease, generating diffuse and poorly immune-infiltrated tumors.[29,41,42] The combination strategy was effective in the two models used, the XFM (H3-WT) and UC-BL6-C7 (H3-altered), regardless of the molecular phenotype. Although they possess distinct mutational profiles, attributing our *in vivo* observations about their different tumor growth kinetics and treatment sensitivity to their mutational characteristics is challenging in this context because they are two separate transplantable models derived from different mouse backgrounds. An aspect to highlight is the difference in the effectiveness of Delta-24-RGD as a single agent compared to our previous work, where tumor cells were injected in the supratentorial region of the brain,[19] and not in the brainstem (specifically in the pons), as is the case now. In that particular area, the tumors exhibit a higher baseline infiltration of immune cells compared to those located in the pons. This increased immune presence may enhance the anti-tumor effect of Delta-24-RGD. Indeed, in a later article where we evaluated the impact of a virus armed with a CD137 ligand (a positive activator of the immune response) and used Delta-24-RGD as a comparison, we found very similar results to those presented in this manuscript.[28] In addition, it is evident that the single treatment with the virus already triggers a potent T recruitment and activation; however, it is not enough to elicit a therapeutic effect. Thus, adding the anti-CD40 leads to a more robust and long-lasting response. An experimental limitation we encounter is that adenoviruses are species specific and that adenovirus serotype 5 (the serotype of the Delta-24-RGD) does not replicate in murine cells.[43] This implies that we only have one round of viruses generating the immune response. In this context, activating DCs through CD40 promotes a sustained anti-tumor

(F) The concentration of the indicated proinflammatory chemokines and cytokines in the tumor microenvironment 6 days after treatment was assessed by LEGENDplex.
(G) Volcano plot showing the differentially expressed genes (assessed by RNA-seq) in XFM tumors treated with the combination compared to IgG control at day 6.
(H) Gene set enrichment analysis of pathways involved in myeloid cell activation and differentiation present in Delta-24-RGD+anti-CD40-treated tumors compared to IgG control and anti-CD40 alone.
(I) Mean of fluorescence intensity (MFI) of MHC-II on microglia and tumor macrophages was measured by flow cytometry 6 days after treatment.
(J) Number of DCs expressing high levels of MHC-II molecules per tumor mg.
(K) Number of tumor DCs expressing CD40 and CD86 per tumor mg.
(L) MFI of PDL1 present on the membrane of tumor DCs at day 6 post-treatment.
(M) Representative example of flow cytometry plots showing the mCherry signal in the indicated populations infiltrating the NP53-mCherry orthotopic DMG model.
(N) Quantification of the mCherry-positive cells from (E) in the tumor niche 24 h after the indicated treatments.
(O) Survival curve of Batf3ko mice bearing UC-BL6-C7 tumor and treated with either the combination or an IgG control.
(P) Representative H&E images of two brains treated with the IgG control and the combination that were collected at the endpoint.
Error bars represent mean ± SEM. One-way ANOVA and the Kruskal-Wallis test were used for statistical analyses from (A)–(H) data. $n = 3–10$ mice per group. Log rank test was used for statistical analysis comparing the combination with IgG control-treated Batf3ko mice in the survival experiments. $n = 4$ mice per group. (*$p < 0.05$; **$p < 0.01$; ***$p < 0.001$; ****$p < 0.0001$).

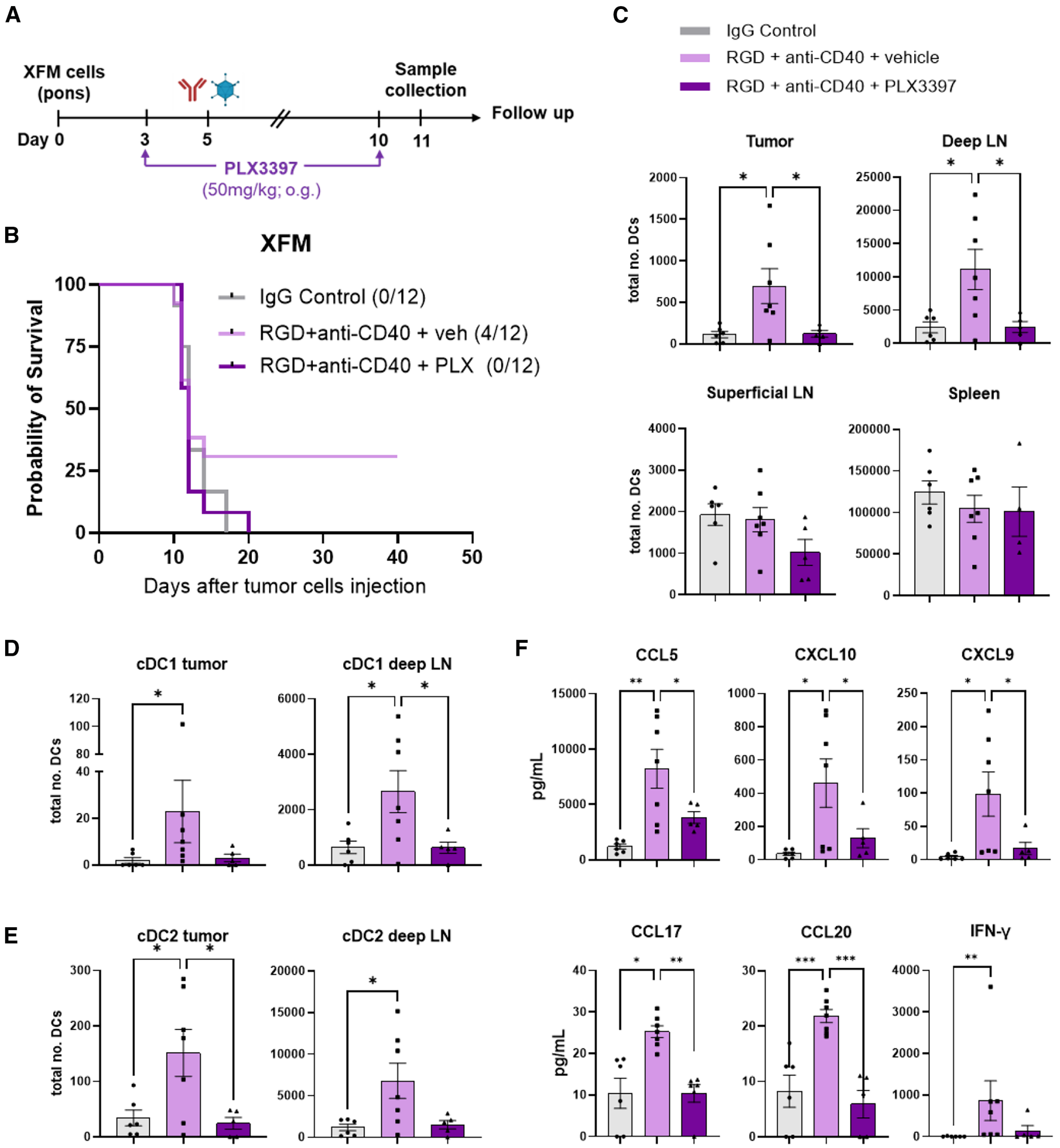

Figure 5. The inhibition of CSF1R avoids the generation of complete responses upon the combination therapy and the recruitment of DCs into the tumor and draining lymph nodes

(A) Experimental overview of the syngeneic model of DMG. 1,000 XFM cells were injected into the pons of BALB/c mice. At day 3, mice were treated via oral gavage with the CSF1R inhibitor (PLX3397, 50 μg/g). On day 5, mice were treated intratumorally with the combination therapy.

(B) Survival curves of XFM-bearing mice treated with the combination in the presence or absence of the CSF1R inhibitor.

(C) Analysis of DC infiltration in XFM tumors, cervical deep and superficial lymph nodes, and spleen 6 days post-treatment.

(D and E) Numbers of cDC1 and cDC2 present in the tumor and deep LN upon RGD+anti-CD40 with or without PLX3397.

(F) Concentration of the indicated proinflammatory chemokines and cytokines in the tumor microenvironment 6 days post-treatment with or without CSF1R inhibition.

LN, lymph nodes. cDC1 and cDC2, conventional dendritic cell type 1 and type 2. Log rank test was used for statistical analysis comparing the combination with IgG control-treatedmice in the survival experiments in (B). The fraction of complete responses per treatment is indicated in brackets. $n = 12$ mice per group. One-way ANOVA and the Kruskal-Wallis test were used for statistical analyses of (C)–(F) data. $n = 6$–7 samples per group. Error bars represent mean ± SEM. (*$p < 0.05$; **$p < 0.01$; ***$p < 0.001$).

T cell response, as indicated by enhanced T cell proliferation. This occurs despite the fact that we did not observe any differences in T cell activation markers when compared to the virus alone.

We have demonstrated that targeting CD40, in addition to Delta-24-RGD, generates durable anti-tumor immunity. Even though we noticed that the peripheral immune response is primarily directed against the virus, the ability of the long-term responder mice to eliminate the rechallenge suggests the development of anti-tumor immunological memory, at least locally, as others have reported.[44] Whether repeated administrations of anti-CD40 could improve the long-term anti-tumor effect must be further studied. As mentioned earlier, the limitation of using mouse syngeneic models is that most of the effect of the virus is due to the activation of the immune system rather than its oncolytic properties since it is not replicative in murine cells.[43] Therefore, we cannot correctly assess the kinetics and the additional benefit of continuously activating the DCs after the virus in our immunocompetent models.

Pediatric DMG patients undergo radiotherapy as the standard of care. In this sense, we have shown that the intratumoral infusion of the Delta-24-RGD followed by radiotherapy in preclinical models of pediatric high-grade glioma (pHGG) and DIPG led to superior survival due to the ability of the adenovirus to downregulate pivotal DNA damage repair proteins, thus sensitizing the glioma cells to the radiotherapy without deleterious effect to the viral replication. Additionally, this combination regimen also fostered a proinflammatory environment.[45] These data were further confirmed in the context of a phase 1 clinical trial where DIPG patients received a single virus injection followed by radiotherapy (NCT03178032). We observed a change in the tumor immune compartment and reduced or stabilized tumor size in some patients.[18] All these data suggest that the generation of pathogenic signals from the virus, together with the danger ones from the cytotoxicity exerted by the virus and the radiotherapy, could feed and foster the effect of DCs, previously activated by a CD40 agonist antibody. To our knowledge, there is only a phase 1 clinical trial testing a humanized anti-CD40 antibody administered systemically for pediatric central nervous system tumors, including newly diagnosed DIPG (NCT03389802). Preliminary data showed that the treatment was, in general, well tolerated. However, three out of 11 DIPG patients presented grade 3 adverse events attributable to the therapy.[46] In addition to the toxicity commonly associated with anti-CD40 agonists, such as cytokine release or hepatotoxicity,[47,48] the systemic administration of the antibody may result in the impossibility of crossing the blood-brain barrier.[49] Thus, in our study, we chose the intratumor administration to overcome any safety and delivery issues and ensure the presence of the antibody in the tumor. In addition, we previously demonstrated that a TIM3 therapeutic antibody quickly drains from the DMG tumor into the draining lymph nodes.[50] Even so, further studies are needed to establish whether the activation of the CD40 receptor is required at the tumor site, the draining lymph nodes, or both. Nevertheless, considering the results from both clinical trials and our safety and efficacy preclinical data, we believe that the administration of the anti-CD40 is feasible and could improve the response to Delta-24-RGD in DMG patients, as explained earlier. In addition, the intratumoral delivery of the virus and CD40 agonist would be the most effective and, as mentioned before, circumvent systemic toxicities. In a potential translation, systemic treatment is the easiest but not necessarily the most effective. Specifically, in the case of DMGs, where other alternative routes of administration have been evaluated in the clinic, such as convention enhanced delivery, it would be feasible to use this local (intratumoral administration) method to achieve superior efficacy.[51,52]

In accordance with the data from other groups that focused on CD40, we found that the anti-tumor effect of the combination is dependent on the DCs, mainly on the cross-presenting DC (cDC1).[53] Indeed, it has been shown that expanding cDC1 improves oncolytic virotherapy and is essential for long-term anti-tumor immune responses in glioblastoma.[54] Mechanistically, we observed an increase in the tumor immune infiltrate mainly due to the inflammatory effect of the virus.[19] However, the addition of the anti-CD40 causes a reorganization of the myeloid compartment by increasing the number of mature DCs in the tumor and the levels of MHC-II molecules in other APCs, such as macrophages and microglia. Transcriptomic data showed that the combination promotes pathways related to antigen presentation, macrophage and DC maturation, and enrichment in CD40 signaling, corroborating the previous data. Delta-24-RGD, as an oncolytic virus, triggers an early proinflammatory response that may lead to the release of tumor antigens.[55] This, in the presence of a CD40 agonist, may explain why DCs have an improved ability to uptake tumor antigens compared to other APCs in DMG-bearing mice. Indeed, this result supports what others have reported about the crucial role of DCs, and not macrophages, in priming tumor antigen-specific T cell responses in brain tumors.[56,57]

Still, we demonstrated that macrophages and microglia play an essential role in the anti-tumor effect of the combination by favoring the recruitment of DCs into the tumor. The CSFR1 inhibitor PLX3397 avoids the presence of cytokines and chemokines at the tumor site in Delta-24-RGD+anti-CD40-treated mice. Some of them, such as CCL5, CXCL10, and CXCL9, are produced by DCs upon activation to favor tumor T cell recruitment.[54] Supporting our data, spleen macrophages, through the CSFR1, are crucial to maintaining the DC pool, emphasizing the importance of resident myeloid cells in regulating the immune microenvironment.[58] In addition, we cannot ignore the recently reported impact of pexidartinib on the differentiation of DCs.[59] Nonetheless, this effect would continue underscoring the importance of these cells in the anti-tumor efficacy of the combination. Unlike with PLX3397, we observed consistent positive responses with the combination. However, administering the vehicle resulted in losing differences in survival rates. One possible explanation is that the mice with DMG are particularly frail at that time. This could imply that the daily oral gavage administration is more intense in this model than in others, potentially affecting the overall condition of the animals.

Our data collectively support a shift in current immunotherapeutic approaches for DMG. We propose initially targeting the tumor myeloid compartment instead of the lymphoid compartment to promote the oncolytic effect of the Delta-24-RGD virus. Our results suggest that enhancing proinflammatory myeloid

cells will ensure the generation of strong adaptive anti-DMG immune responses.

Limitations of the study

While advances in translational therapy for DMG are encouraging, our study presents some limitations. First, employing immunocompetent models is critical for understanding the role of the immune system in the anti-tumor efficacy. Nonetheless, the replication of the Delta-24-RGD oncolytic virus in murine tumor cells is significantly impeded. Thus, the therapeutic effects observed are predominantly mediated by immune activation rather than by the direct oncolytic activity of the virus. To facilitate such an oncolytic effect in our models, we must conduct experiments on immunodeficient mice harboring human xenograft tumors where the virus replicates. However, the study of the immune response to CD40 activation is limited in this setting. Repeated administrations could potentially mimic, at least partially, the replication of Delta-24-RGD in an immunocompetent context, thereby addressing both limitations. Mechanistically, the contribution of microglia and tumor-associated macrophages to the therapeutic efficacy remains to be fully elucidated.

RESOURCE AVAILABILITY

Lead contact

Requests for further information, resources, and reagents should be directed to and will be fulfilled by the lead contact, Marta M. Alonso (mmalonso@unav.es).

Materials availability

All reagents generated in this study are available from the lead contact with a completed material transfer agreement.

Data and code availability

RNA-seq data have been deposited in GEO (GSE292938) and are publicly available as of the date of publication. The accession number is listed in the key resources table. This study did not generate new original code. Any additional information required to reanalyze the data reported in this work paper is available from the lead contact upon request.

ACKNOWLEDGMENTS

The performed work was supported through the PCI2021-122084-2B from MICIU/AEI/10.13039/501100011033 and, as appropriate, by the “European Union NextGenerationEU/PRTR” (S.L. and M.M.A.), Alicia Koplowitz Foundation “Grant for research in Psychiatry and Neuroscience in Children and Adolescents 2021” (S.L. and M.M.A.), Blanca Morell Foundation “Research Projects in Diffuse Midline Gliomas 2021” (J.G.P.-L. and S.L.), CRIS Contra el Cáncer Foundation “CRIS Emerging Leader Program 2024” (S.L.), GRANATE project funded by the Government of Navarre in the frame of “Proyectos Estratégicos de I+D 2022–2025 Reto GEMA (001-1411-2022-000066)” (M.M.A., A.P.-G., and S.L.), ChadTough-Defeat DIPG (M.M.A.), AECC General Projects (PRYGN21937; M.M.A.), Instituto de Salud Carlos III y Fondos Feder (PI19/01896 MMA, PI20-01132 FP, and PI18/00164 APG “A way to make Europe”), Fundación El Sueño de Vicky, Asociación Pablo Ugarte-Fuerza Julen, Fundación ADEY, Fundación ACS, Cambiando vidas con Laura (A.P.-G. and M.M.A.), Fundación Hay que tomarse la vida con tumor, Fundación + Investigación + Vida (La Guareña), and Red Española de Terapias Avanzadas TERAV ISCIII (RD21/0001/0022; funded by European Union NextGenerationEU/PRTR). This project also received funding from the European Research Council (ERC) under the European Union’s Horizon 2020 Research and Innovation Programme (817884 ViroPedTher to M.M.A.), Predoctoral Fellowships from Gobierno de Navarra (V.L.), Instituto de Salud Carlos III (D.d.I.N.), and ChadTough-Defeat DIPG (R.H.-O.).

AUTHOR CONTRIBUTIONS

Conception and design: S.L. and M.M.A. Development of methodology: all authors. Acquisition of data (provided animals, acquired and managed patients, provided facilities, etc.): all authors. Analysis and interpretation of the data (e.g., statistical analysis, biostatistics, and computational analysis): all authors. Writing, review, and/or revision of the manuscript: all authors. Administrative, technical, or material support (i.e., reporting or organizing data and constructing databases): all authors. Study supervision: S.L. and M.M.A.

DECLARATION OF INTERESTS

M.M.A. and J.G.P.-L. have a patent application PCT/US2022/026392 (Use of oncolytic adenovirus for the treatment of pediatric brain cancer) filed by the host institution and DNAtrix on April 26, 2022 (currently in prosecution in Europe and the USA).

STAR★METHODS

Detailed methods are provided in the online version of this paper and include the following:

- KEY RESOURCES TABLE
- EXPERIMENTAL MODEL AND STUDY PARTICIPANT DETAILS
  - Mice
  - Cell lines
- METHOD DETAILS
  - Tumor cell injection and *in vivo* treatments
  - Cell isolation and flow cytometry assays
  - H&E and immunofluorescence
  - LegendPlex
  - ELISPOT assays
  - cDC1 differentiation
  - *In vitro* DC phagocytosis, maturation, and antigen presentation assays
  - RNAseq
- QUANTIFICATION AND STATISTICAL ANALYSES

SUPPLEMENTAL INFORMATION

Supplemental information can be found online at https://doi.org/10.1016/j.xcrm.2025.102204.

Received: October 8, 2024
Revised: March 28, 2025
Accepted: May 30, 2025
Published: June 26, 2025

REFERENCES

1. Saratsis, A.M., Knowles, T., Petrovic, A., and Nazarian, J. (2024). H3K27M mutant glioma: Disease definition and biological underpinnings. https://doi.org/10.1093/neuonc/noad164.
2. Ebrahimi, A., Skardelly, M., Schuhmann, M.U., Ebinger, M., Reuss, D., Neumann, M., Tabatabai, G., Kohlhof-Meinecke, P., and Schittenhelm, J. (2019). High frequency of H3 K27M mutations in adult midline gliomas. J. Cancer Res. Clin. Oncol. *145*, 839–850. https://doi.org/10.1007/s00432-018-02836-5.
3. Al Sharie, S., Abu Laban, D., and Al-Hussaini, M. (2023). Decoding Diffuse Midline Gliomas: A Comprehensive Review of Pathogenesis, Diagnosis and Treatment. https://doi.org/10.3390/cancers15194869.
4. Cooney, T., Lane, A., Bartels, U., Bouffet, E., Goldman, S., Leary, S.E.S., Foreman, N.K., Packer, R.J., Broniscer, A., Minturn, J.E., et al. (2017).

CellPress
OPEN ACCESS

Contemporary survival endpoints: An international diffuse intrinsic pontine glioma registry study. https://doi.org/10.1093/neuonc/nox107.

5. Van Den Bent, M., Saratsis, A.M., Geurts, M., and Franceschi, E. (2024). H3 K27M-Altered glioma and diffuse intrinsic pontine glioma: Semi-systematic review of treatment landscape and future directions. https://doi.org/10.1093/neuonc/noad220.
6. Cossarizza, A., Chang, H.-D., Radbruch, A., Acs, A., Adam, D., Adam-Klages, S., Agace, W.W., Aghaeepour, N., Akdis, M., Allez, M., et al. (2019). Guidelines for the use of flow cytometry and cell sorting in immunological studies (second edition). Eur. J. Immunol. *49*, 1457–1973. https://doi.org/10.1002/eji.201970107.
7. Lieberman, N.A.P., DeGolier, K., Kovar, H.M., Davis, A., Hoglund, V., Stevens, J., Winter, C., Deutsch, G., Furlan, S.N., Vitanza, N.A., et al. (2019). Characterization of the immune microenvironment of diffuse intrinsic pontine glioma: implications for development of immunotherapy. Neuro Oncol. *21*, 83–94. https://doi.org/10.1093/neuonc/noy145.
8. Liu, I., Jiang, L., Samuelsson, E.R., Marco Salas, S., Beck, A., Hack, O.A., Jeong, D., Shaw, M.L., Englinger, B., LaBelle, J., et al. (2022). The landscape of tumor cell states and spatial organization in H3-K27M mutant diffuse midline glioma across age and location. Nat. Genet. *54*, 1881–1894. https://doi.org/10.1038/s41588-022-01236-3.
9. Persson, M.L., Douglas, A.M., Alvaro, F., Faridi, P., Larsen, M.R., Alonso, M.M., Vitanza, N.A., and Dun, M.D. (2022). The intrinsic and microenvironmental features of diffuse midline glioma: Implications for the development of effective immunotherapeutic treatment strategies. https://doi.org/10.1093/neuonc/noac117.
10. Kline, C., Liu, S.J., Duriseti, S., Banerjee, A., Nicolaides, T., Raber, S., Gupta, N., Haas-Kogan, D., Braunstein, S., and Mueller, S. (2018). Reirradiation and PD-1 inhibition with nivolumab for the treatment of recurrent diffuse intrinsic pontine glioma: a single-institution experience. J. Neuro Oncol. *140*, 629–638. https://doi.org/10.1007/s11060-018-2991-5.
11. Cacciotti, C., Choi, J., Alexandrescu, S., Zimmerman, M.A., Cooney, T.M., Chordas, C., Clymer, J., Chi, S., and Yeo, K.K. (2020). Immune checkpoint inhibition for pediatric patients with recurrent/refractory CNS tumors: a single institution experience. J. Neuro Oncol. *149*, 113–122. https://doi.org/10.1007/s11060-020-03578-6.
12. Mount, C.W., Majzner, R.G., Sundaresh, S., Arnold, E.P., Kadapakkam, M., Haile, S., Labanieh, L., Hulleman, E., Woo, P.J., Rietberg, S.P., et al. (2018). Potent antitumor efficacy of anti-GD2 CAR T cells in H3-K27M(+) diffuse midline gliomas. Nat. Med. *24*, 572–579. https://doi.org/10.1038/s41591-018-0006-x.
13. Theruvath, J., Sotillo, E., Mount, C.W., Graef, C.M., Delaidelli, A., Heitzeneder, S., Labanieh, L., Dhingra, S., Leruste, A., Majzner, R.G., et al. (2020). Locoregionally administered B7-H3-targeted CAR T cells for treatment of atypical teratoid/rhabdoid tumors. Nat. Med. *26*, 712–719. https://doi.org/10.1038/s41591-020-0821-8.
14. Majzner, R.G., Ramakrishna, S., Yeom, K.W., Patel, S., Chinnasamy, H., Schultz, L.M., Richards, R.M., Jiang, L., Barsan, V., Mancusi, R., et al. (2022). GD2-CAR T cell therapy for H3K27M-mutated diffuse midline gliomas. Nature *603*, 934–941. https://doi.org/10.1038/s41586-022-04489-4.
15. Vitanza, N.A., Wilson, A.L., Huang, W., Seidel, K., Brown, C., Gustafson, J.A., Yokoyama, J.K., Johnson, A.J., Baxter, B.A., Koning, R.W., et al. (2023). Intraventricular B7-H3 CAR T cells for diffuse intrinsic pontine glioma: preliminary first-in-human bioactivity and safety. Cancer Discov. *13*, 114–131. https://doi.org/10.1158/2159-8290.CD-22-0750.
16. Suzuki, K., Fueyo, J., Krasnykh, V., Reynolds, P.N., Curiel, D.T., and Alemany, R. (2001). A conditionally replicative adenovirus with enhanced infectivity shows improved oncolytic potency. Clin. Cancer Res. *7*, 120–126.
17. Fueyo, J., Alemany, R., Gomez-Manzano, C., Fuller, G.N., Khan, A., Conrad, C.A., Liu, T.J., Jiang, H., Lemoine, M.G., Suzuki, K., et al. (2003). Preclinical characterization of the antiglioma activity of a tropism-enhanced adenovirus targeted to the retinoblastoma pathway. J. Natl. Cancer Inst. *95*, 652–660. https://doi.org/10.1093/jnci/95.9.652.
18. Gállego Pérez-Larraya, J., Garcia-Moure, M., Labiano, S., Patiño-García, A., Dobbs, J., Gonzalez-Huarriz, M., Zalacain, M., Marrodan, L., Martinez-Velez, N., Puigdelloses, M., et al. (2022). Oncolytic DNX-2401 Virus for Pediatric Diffuse Intrinsic Pontine Glioma. N. Engl. J. Med. *386*, 2471–2481. https://doi.org/10.1056/NEJMoa2202028.
19. Martínez-Vélez, N., Garcia-Moure, M., Marigil, M., González-Huarriz, M., Puigdelloses, M., Gallego Pérez-Larraya, J., Zalacaín, M., Marrodán, L., Varela-Guruceaga, M., Laspidea, V., et al. (2019). The oncolytic virus Delta-24-RGD elicits an antitumor effect in pediatric glioma and DIPG mouse models. Nat. Commun. *10*, 2235. https://doi.org/10.1038/s41467-019-10043-0.
20. Yang, K., Halima, A., and Chan, T.A. (2023). Antigen presentation in cancer — mechanisms and clinical implications for immunotherapy. https://doi.org/10.1038/s41571-023-00789-4.
21. Sun, Y.Y., Peng, S., Han, L., Qiu, J., Song, L., Tsai, Y., Yang, B., Roden, R.B.S., Trimble, C.L., Hung, C.F., and Wu, T.C. (2016). Local HPV Recombinant Vaccinia Boost Following Priming with an HPV DNA Vaccine Enhances Local HPV-Specific CD8+T-cell-Mediated Tumor Control in the Genital Tract. Clin. Cancer Res. *22*, 657–669. https://doi.org/10.1158/1078-0432.CCR-15-0234.
22. Byrne, K.T., and Vonderheide, R.H. (2016). CD40 Stimulation Obviates Innate Sensors and Drives T Cell Immunity in Cancer. Cell Rep. *15*, 2719–2732. https://doi.org/10.1016/j.celrep.2016.05.058.
23. Buhtoiarov, I.N., Sondel, P.M., Wigginton, J.M., Buhtoiarova, T.N., Yanke, E.M., Mahvi, D.A., and Rakhmilevich, A.L. (2011). Anti-tumour synergy of cytotoxic chemotherapy and anti-CD40 plus CpG-ODN immunotherapy through repolarization of tumour-associated macrophages. Immunology *132*, 226–239. https://doi.org/10.1111/j.1365-2567.2010.03357.x.
24. Mathys, H., Adaikkan, C., Gao, F., Young, J.Z., Manet, E., Hemberg, M., De Jager, P.L., Ransohoff, R.M., Regev, A., and Tsai, L.H. (2017). Temporal Tracking of Microglia Activation in Neurodegeneration at Single-Cell Resolution. Cell Rep. *21*, 366–380. https://doi.org/10.1016/j.celrep.2017.09.039.
25. Beatty, G.L., Chiorean, E.G., Fishman, M.P., Saboury, B., Teitelbaum, U.R., Sun, W., Huhn, R.D., Song, W., Li, D., Sharp, L.L., et al. (2011). CD40 agonists alter tumor stroma and show efficacy against pancreatic carcinoma in mice and humans. Science *331*, 1612–1616. https://doi.org/10.1126/science.1198443.
26. Shoji, T., Saito, R., Chonan, M., Shibahara, I., Sato, A., Kanamori, M., Sonoda, Y., Kondo, T., Ishii, N., and Tominaga, T. (2016). Local convection-enhanced delivery of an anti-CD40 agonistic monoclonal antibody induces antitumor effects in mouse glioma models. Neuro Oncol. *18*, 1120–1128. https://doi.org/10.1093/neuonc/now023.
27. Messenheimer, D.J., Jensen, S.M., Afentoulis, M.E., Wegmann, K.W., Feng, Z., Friedman, D.J., Gough, M.J., Urba, W.J., and Fox, B.A. (2017). Timing of PD-1 Blockade Is Critical to Effective Combination Immunotherapy with Anti-OX40. Clin. Cancer Res. *23*, 6165–6177. https://doi.org/10.1158/1078-0432.CCR-16-2677.
28. Laspidea, V., Puigdelloses, M., Labiano, S., Marrodán, L., Garcia-Moure, M., Zalacain, M., Gonzalez-Huarriz, M., Martínez-Vélez, N., Ausejo-Mauleon, I., de la Nava, D., et al. (2022). Exploiting 4-1BB immune checkpoint to enhance the efficacy of oncolytic virotherapy for diffuse intrinsic pontine gliomas. JCI Insight *7*, e154812. https://doi.org/10.1172/jci.insight.154812.
29. Du Chatinier, A., Meel, M.H., Das, A.I., Metselaar, D.S., Waranecki, P., Bugiani, M., Breur, M., Simonds, E.F., Lu, E.D., Weiss, W.A., et al. (2022). Generation of immunocompetent syngeneic allograft mouse models for pediatric diffuse midline glioma. Neurooncol. Adv. *4*, vdac079. https://doi.org/10.1093/noajnl/vdac079.
30. Peng, Q., Qiu, X., Zhang, Z., Zhang, S., Zhang, Y., Liang, Y., Guo, J., Peng, H., Chen, M., Fu, Y.-X., and Tang, H. (2020). PD-L1 on dendritic cells attenuates T cell activation and regulates response to immune checkpoint blockade. Nat. Commun. *11*, 4835. https://doi.org/10.1038/s41467-020-18570-x.

31. Koschmann, C., Al-Holou, W.N., Alonso, M.M., Anastas, J., Bandopadhayay, P., Barron, T., Becher, O., Cartaxo, R., Castro, M.G., Chung, C., et al. (2024). A road map for the treatment of pediatric diffuse midline glioma. Cancer Cell *42*, 1–5. https://doi.org/10.1016/j.ccell.2023.11.002.

32. Lin, G.L., Nagaraja, S., Filbin, M.G., Suvà, M.L., Vogel, H., and Monje, M. (2018). Non-inflammatory tumor microenvironment of diffuse intrinsic pontine glioma. Acta Neuropathol. Commun. *6*, 51. https://doi.org/10.1186/s40478-018-0553-x.

33. Foster, J.B., Madsen, P.J., Hegde, M., Ahmed, N., Cole, K.A., Maris, J.M., Resnick, A.C., Storm, P.B., and Waanders, A.J. (2019). Immunotherapy for pediatric brain tumors: past and present. Neuro Oncol. *21*, 1226–1238. https://doi.org/10.1093/neuonc/noz077.

34. Goswami, S., Anandhan, S., Raychaudhuri, D., and Sharma, P. (2023). Myeloid cell-targeted therapies for solid tumours. Nat. Rev. Immunol. *23*, 106–120. https://doi.org/10.1038/s41577-022-00737-w.

35. Musher, B.L., Rowinsky, E.K., Smaglo, B.G., Abidi, W., Othman, M., Patel, K., Jawaid, S., Jing, J., Brisco, A., Leen, A.M., et al. (2024). LOAd703, an oncolytic virus-based immunostimulatory gene therapy, combined with chemotherapy for unresectable or metastatic pancreatic cancer (LOKON001): results from arm 1 of a non-randomised, single-centre, phase 1/2 study. Lancet Oncol. *25*, 488–500. https://doi.org/10.1016/S1470-2045(24)00079-2.

36. Medina-Echeverz, J., Hinterberger, M., Testori, M., Geiger, M., Giessel, R., Bathke, B., Kassub, R., Gräbnitz, F., Fiore, G., Wennier, S.T., et al. (2019). Synergistic cancer immunotherapy combines MVA-CD40L induced innate and adaptive immunity with tumor targeting antibodies. Nat. Commun. *10*, 5041. https://doi.org/10.1038/s41467-019-12998-6.

37. Ylösmäki, E., Ylösmäki, L., Fusciello, M., Martins, B., Ahokas, P., Cojoc, H., Uoti, A., Feola, S., Kreutzman, A., Ranki, T., et al. (2021). Characterization of a novel OX40 ligand and CD40 ligand-expressing oncolytic adenovirus used in the PeptiCRAd cancer vaccine platform. Mol. Ther. Oncolytics *20*, 459–469. https://doi.org/10.1016/j.omto.2021.02.006.

38. Pesonen, S., Diaconu, I., Kangasniemi, L., Ranki, T., Kanerva, A., Pesonen, S.K., Gerdemann, U., Leen, A.M., Kairemo, K., Oksanen, M., et al. (2012). Oncolytic immunotherapy of advanced solid tumors with a CD40L-expressing replicating adenovirus: Assessment of safety and immunologic responses in patients. Cancer Res. *72*, 1621–1631. https://doi.org/10.1158/0008-5472.CAN-11-3001.

39. Puigdelloses, M., Garcia-Moure, M., Labiano, S., Laspidea, V., Gonzalez-Huarriz, M., Zalacain, M., Marrodan, L., Martinez-Velez, N., De La Nava, D., Ausejo, I., et al. (2021). CD137 and PD-L1 targeting with immunovirotherapy induces a potent and durable antitumor immune response in glioblastoma models. J. Immunother. Cancer *9*, e002644. https://doi.org/10.1136/jitc-2021-002644.

40. Wongthida, P., Schuelke, M.R., Driscoll, C.B., Kottke, T., Thompson, J.M., Tonne, J., Stone, C., Huff, A.L., Wetmore, C., Davies, J.A., et al. (2020). Ad-CD40L mobilizes CD4 T cells for the treatment of brainstem tumors. Neuro Oncol. *22*, 1757–1770. https://doi.org/10.1093/neuonc/noaa126.

41. Halvorson, K.G., Barton, K.L., Schroeder, K., Misuraca, K.L., Hoeman, C., Chung, A., Crabtree, D.M., Cordero, F.J., Singh, R., Spasojevic, I., et al. (2015). A high-throughput in Vitro drug screen in a genetically engineered mouse model of diffuse intrinsic pontine glioma identifies BMS-754807 as a promising therapeutic agent. PLoS One *10*, e0118926. https://doi.org/10.1371/journal.pone.0118926.

42. Barton, K.L., Misuraca, K., Cordero, F., Dobrikova, E., Min, H.D., Gromeier, M., Kirsch, D.G., and Becher, O.J. (2013). PD-0332991, a CDK4/6 Inhibitor, Significantly Prolongs Survival in a Genetically Engineered Mouse Model of Brainstem Glioma. PLoS One *8*, e77639. https://doi.org/10.1371/journal.pone.0077639.

43. Blair, G.E., Dixon, S.C., Griffiths, S.A., and Zajdel, M.E. (1989). Restricted replication of human adenovirus type 5 in mouse cell lines. Virus Res. *14*, 339–346. https://doi.org/10.1016/0168-1702(89)90026-9.

44. Thompson, E.A., Liang, F., Lindgren, G., Sandgren, K.J., Quinn, K.M., Darrah, P.A., Koup, R.A., Seder, R.A., Kedl, R.M., and Loré, K. (2015). Human Anti-CD40 Antibody and Poly IC:LC Adjuvant Combination Induces Potent T Cell Responses in the Lung of Nonhuman Primates. J. Immunol. *195*, 1015–1024. https://doi.org/10.4049/jimmunol.1500078.

45. Martinez-Velez, N., Marigil, M., García-Moure, M., Gonzalez-Huarriz, M., Aristu, J.J., Ramos-García, L.I., Tejada, S., Díez-Valle, R., Patiño-García, A., Becher, O.J., et al. (2019). Delta-24-RGD combined with radiotherapy exerts a potent antitumor effect in diffuse intrinsic pontine glioma and pediatric high grade glioma models. Acta Neuropathol. Commun. *7*, 64. https://doi.org/10.1186/S40478-019-0714-6.

46. Lindsay, H.B., Billups, C., Baxter, P., Hwang, E., Lenzen, A., MacDonald, T., Pollack, I.F., Robison, N., Vinitsky, A., Poussaint, T.Y., et al. (2024). TRLS-06. PHASE 1 STUDY OF THE CD40 AGONISTIC MONOCLONAL ANTIBODY APX005M (SOTIGALIMAB) IN PEDIATRIC, ADOLESCENT, AND YOUNG ADULT PATIENTS WITH RECURRENT OR PROGRESSIVE CNS TUMORS AND NEWLY DIAGNOSED BRAIN STEM GLIOMA: A PEDIATRIC BRAIN TUMOR CONSORTIUM STUDY (PBTC-051; NCT03389802). Neuro Oncol. *26*. https://doi.org/10.1093/neuonc/noae064.159.

47. Vonderheide, R.H., and Glennie, M.J. (2013). Agonistic CD40 antibodies and cancer therapy. Clin. Cancer Res. *19*, 1035–1043. https://doi.org/10.1158/1078-0432.CCR-12-2064.

48. Bonnans, C., Thomas, G., He, W., Jung, B., Chen, W., Liao, M., Heyen, J., Buetow, B., Pillai, S., Matsumoto, D., et al. (2020). CD40 agonist-induced IL-12p40 potentiates hepatotoxicity. J. Immunother. Cancer *8*, e000624. https://doi.org/10.1136/jitc-2020-000624.

49. Zuchero, Y.J.Y., Chen, X., Bien-Ly, N., Bumbaca, D., Tong, R.K., Gao, X., Zhang, S., Hoyte, K., Luk, W., Huntley, M.A., et al. (2016). Discovery of Novel Blood-Brain Barrier Targets to Enhance Brain Uptake of Therapeutic Antibodies. Neuron *89*, 70–82. https://doi.org/10.1016/j.neuron.2015.11.024.

50. Ausejo-Mauleon, I., Labiano, S., de la Nava, D., Laspidea, V., Zalacain, M., Marrodán, L., García-Moure, M., González-Huarriz, M., Hervás-Corpión, I., Dhandapani, L., et al. (2023). TIM-3 blockade in diffuse intrinsic pontine glioma models promotes tumor regression and antitumor immune memory. Cancer Cell *41*, 1911–1926.e8. https://doi.org/10.1016/j.ccell.2023.09.001.

51. Giantini-Larsen, A.M., Pandey, A., Garton, A.L.A., Rampichini, M., Winston, G., Goldberg, J.L., Magge, R., Stieg, P.E., Souweidane, M.M., and Ramakrishna, R. (2025). Therapeutic manipulation and bypass of the blood–brain barrier: powerful tools in glioma treatment. Neurooncol. Adv. *7*, vdae201. https://doi.org/10.1093/noajnl/vdae201.

52. Souweidane, M.M., Bander, E.D., Zanzonico, P., Reiner, A.S., Manino, N., Haque, S., Carrasquillo, J.A., Lyashchenko, S.K., Thakur, S.B., Lewis, J. S., et al. (2025). Phase 1 dose-escalation trial using convection-enhanced delivery (CED) of radio-immunotheranostic 124I-Omburtamab for diffuse intrinsic pontine glioma (DIPG). Neuro Oncol. noaf039. https://doi.org/10.1093/neuonc/noaf039.

53. Ferris, S.T., Durai, V., Wu, R., Theisen, D.J., Ward, J.P., Bern, M.D., Davidson, J.T., Bagadia, P., Liu, T., Briseño, C.G., et al. (2020). cDC1 prime and are licensed by CD4+ T cells to induce anti-tumour immunity. Nature *584*, 624–629. https://doi.org/10.1038/s41586-020-2611-3.

54. Svensson-Arvelund, J., Cuadrado-Castano, S., Pantsulaia, G., Kim, K., Aleynick, M., Hammerich, L., Upadhyay, R., Yellin, M., Marsh, H., Oreper, D., et al. (2022). Expanding cross-presenting dendritic cells enhances oncolytic virotherapy and is critical for long-term anti-tumor immunity. Nat. Commun. *13*, 7149. https://doi.org/10.1038/s41467-022-34791-8.

55. Lin, D., Shen, Y., and Liang, T. (2023). Oncolytic virotherapy: basic principles, recent advances and future directions. https://doi.org/10.1038/s41392-023-01407-6.

56. Malo, C.S., Huggins, M.A., Goddery, E.N., Tolcher, H.M.A., Renner, D.N., Jin, F., Hansen, M.J., Pease, L.R., Pavelko, K.D., and Johnson, A.J. (2018). Non-equivalent antigen presenting capabilities of dendritic cells and macrophages in generating brain-infiltrating CD8 + T cell responses. Nat. Commun. *9*, 633. https://doi.org/10.1038/s41467-018-03037-x.

CellPress
OPEN ACCESS

57. Bowman-Kirigin, J.A., Desai, R., Saunders, B.T., Wang, A.Z., Schaettler, M.O., Liu, C.J., Livingstone, A.J., Kobayashi, D.K., Durai, V., Kretzer, N. M., et al. (2023). The Conventional Dendritic Cell 1 Subset Primes CD8+ T Cells and Traffics Tumor Antigen to Drive Antitumor Immunity in the Brain. Cancer Immunol. Res. *11*, 20–37. https://doi.org/10.1158/2326-6066.CIR-22-0098.

58. Percin, G.I., Eitler, J., Kranz, A., Fu, J., Pollard, J.W., Naumann, R., and Waskow, C. (2018). CSF1R regulates the dendritic cell pool size in adult mice via embryo-derived tissue-resident macrophages. Nat. Commun. *9*, 5279. https://doi.org/10.1038/s41467-018-07685-x.

59. Voissière, A., Gomez-Roca, C., Chabaud, S., Rodriguez, C., Nkodia, A., Berthet, J., Montane, L., Bidaux, A.S., Treilleux, I., Eberst, L., et al. (2024). The CSF-1R inhibitor pexidartinib affects FLT3-dependent DC differentiation and may antagonize durvalumab effect in patients with advanced cancers. Sci. Transl. Med. *16*, eadd1834. https://doi.org/10.1126/scitranslmed.add1834.

60. Schneider, C.A., Rasband, W.S., and Eliceiri, K.W. (2012). NIH Image to ImageJ: 25 years of image analysis. Nat. Methods *9*, 671–675. https://doi.org/10.1038/nmeth.2089.

61. Bolger, A.M., Lohse, M., and Usadel, B. (2014). Trimmomatic: a flexible trimmer for Illumina sequence data. Bioinformatics *30*, 2114–2120. https://doi.org/10.1093/bioinformatics/btu170.

62. Ausejo-Mauleon, I., Labiano, S., and Alonso, M.M. (2024). Characterization of immune populations in the tumor microenvironment of diffuse midline glioma orthotopic mouse models by flow cytometry. STAR Protoc. *5*, 102803. https://doi.org/10.1016/j.xpro.2023.102803.

STAR★METHODS

KEY RESOURCES TABLE

REAGENT or RESOURCE	SOURCE	IDENTIFIER
Antibodies		
Anti-mouse CD16/CD32	BioLegend	Cat#101319 ; RRID: AB_1574973
AF700 anti-mouse CD45	BioLegend	Cat#103128; RRID: AB_493714
BUV395 anti-mouse CD11b	BD Bioscience	Cat#563553; RRID: AB_2738276
BUV496 anti-mouse CD4	BD Bioscience	Cat# 612952; RRID: AB_2813886
BV605 anti-mouse NK1.1	BioLegend	Cat# 108739; RRID: AB_2562273
BV510 anti-mouse CD8a	BioLegend	Cat# 100752; RRID: AB_2563057
APC anti-mouse F4/80	BioLegend	Cat# 123116; RRID: AB_893481
BV421 Anti-mouse CD19	BioLegend	Cat# 115538; RRID: AB_11203527
BV785 anti-mouse TCRb	BioLegend	Cat# 109249; RRID: AB_2810347
PE/Cy7 anti-mouse CD11c	BioLegend	Cat# 117318; RRID: AB_493568
BV610 anti-mouse I-A/I-E	BioLegend	Cat# 107641; RRID: AB_2565975
PE anti-mouse SIRPa	BioLegend	Cat# 144005; RRID: AB_11204432
FITC anti-mouse XCR1	BioLegend	Cat# 148210; RRID: AB_2564366
APC/Cy7 anti-mouse XR1	BioLegend	Cat#148223 ; RRID: AB_2783117
PerCP/Cy5.5 anti-mouse Ly6G	BioLegend	Cat# 127616; RRID: AB_1877271
APC-Vio770 Anti-mouse Ly6C (REA796)	Miltenyi Biotec	Cat# 130-111-919
BV510 anti-mouse CD44	BioLegend	Cat# 103044; RRID: AB_2650923
APC anti-mouse CD62L	BioLegend	Cat# 104411; RRID: AB_313098
FOXP3 Monoclonal Antibody, PE-eFluor™ 610	ThermoFisher	Cat# 61-5773-82; RRID: AB_2574624
FITC anti-mouse CD80	BioLegend	Cat# 104705; RRID: AB_313126
PerCP/Cy5.5 anti-mouse CD86	BioLegend	Cat# 105028; RRID: AB_2074994
BV421 anti-mouse CD274	BioLegend	Cat# 135218; RRID: AB_2561447
FITC anti-mouse ki67	BioLegend	Cat# 100510; RRID: AB_312713
PerCP/Cy5.5 anti-mouse IFN-y	BioLegend	Cat#505821 ; RRID: AB_961361
APC anti-mouse TNF-a	BioLegend	Cat#506307; RRID: AB_315428
Granzyme B monoclonal antibody, PE-Cy7	eBioscience	Cat#25-8898-80; RRID: AB_10853338
InVivoMAb anti-mouse CD40	BioXCell	Cat#BE0016-2; RRID: AB_1107647
InVivoMAb rat IgG2a isotype control, anti-trinitrophenol	BioXCell	Cat#BE0089; RRID: AB_1107769
Anti-mouse CD11c	CellSignalling	Cat#97585; RRID:AB_2800282
Goat anti-Rabbit IgG (H+L) Antibody, Alexa Fluor 647	ThermoFisher	Cat#A-21244; RRID: AB_2535812
Bacterial and virus strains		
Adenovirus Delta-24-RGD	Laboratory of Dr. Marta Alonso	N/A
Chemicals, peptides, and recombinant proteins		
LPS	Merck	Cat#L3023
Dexamethasone	Merck	Cat#D4902
FGF human	Merck	Cat#SRP3027
EGF human	Merck	Cat#SRP4037
GM-CSF	Immunotools	Cat#12343122
FLT3L	BioXCell	Cat#BE0342

(*Continued on next page*)

Continued

REAGENT or RESOURCE	SOURCE	IDENTIFIER
PLX3397	MedChemExpress	Cat#HY-16749
Beetle Luciferin Potassium Salt	Promega	Cat#E1601
VPD450	BD Bioscience	Cat#562158
Promofluor 840	DeltaIon	Cat#1402
hgp100	GenScript	Cat#RP20344
FTY700	Sigma-Aldrich	Cat#162359-56-0
**Critical commercial assays**		
Legendplex mouse inflammation	BioLegend	Cat#740150
Legendplex mouse pro-inflammatory chemokine	BioLegend	Cat#740007
IFN-y ELISPOT	BD Bioscience	Cat#551083
RNeasy Mini Kit	Qiagen	Cat#74104
**Deposited data**		
RNAseq raw	This paper	GSE292938
**Experimental models: Cell lines**		
Murine DIPG: XFM	Laboratory of Dr. Oren Becher	N/A
Murine DIPG: NP53-mCherry	Laboratory of Dr. Marta Alonso	N/A
Murine DIPG: XFM-luc	Laboratory of Dr. Marta Alonso	N/A
Murine DIPG: UC-BL6-C7	Laboratory of Dr. Timothy N. Phoenix	N/A
Murine DIPG: UC-BL6-C7-Tomato	Laboratory of Dr. Marta Alonso	N/A
Human: HEK293T	ATCC	CRL-3216
**Experimental models: Organisms/strains**		
Mouse: BALB/cJ	The Jackson Laboratory	JAX: 000651
Mouse: C57Bl/6J	The Jackson Laboratory	JAX: 000664
Mouse: B6.Cg-Thy1a/Cy Tg(TcraTcrb)8Rest/J	The Jackson Laboratory	JAX: 005023
Mouse: B6.129S(C)-Batf3tm1Kmm/J	The Jackson Laboratory	JAX: 013755
**Software and algorithms**		
PhotonIMAGER Optima system	BioscpaceLab	N/A
Aperio ImageScope	Leica Biosystems	N/A
ImageJ	Schneider et al.[60]	https://imagej.nih.gov/ij/
FlowJo 10.8.1	BD Bioscience	N/A
QuPath version 0.2.3	University of Edinburgh	N/A
R/Bioconductor	Bioconductor	N/A
R 3.6.0	Posit	N/A
Legendplex analysis Software V8.0	Vigine Tech Inc	N/A
FastQC version 0.11.8	Babraham Bioinformatics	N/A
Trimmomatic version 0.38	Bolger, Anthony M et al.[61]	N/A
Subread version 1.6.3	Subread	N/A
EdgeR version 3.40.2	Bioconductor	N/A
ClusterProfile R package	Bioconductor	N/A
Prism software 10	GraphPad by Domatics	N/A
BioRender	BioRender	N/A

EXPERIMENTAL MODEL AND STUDY PARTICIPANT DETAILS

Mice

Four-week-old Balb/c and C57BL6/J mice were purchased from Harlan. *Batf3*−/− mice were kindly gifted by Dr. Prieto (University of Navarra, Pamplona). NTV-a p53fl/fl, Rag2-Il2Rγ-ko and B6.Cg-Thy1a/Cy Tg(TcraTcrb)8Rest/J (PMEL) mice were bred in our facilities. For breeding, one male and two females were housed per cage within a ventilated system. The offspring were weaned at three weeks old and transferred to ventilated cages containing up to six mice each. Four- to five-week-old female and male animals were used in the *in vivo* experiments. Mice were housed in groups of six in individually ventilated cages under pathogen-free conditions at

the Center for Applied Medical Research animal facility (Pamplona, Spain). Animal procedures were conducted under license 068-20, approved by the University of Navarra Ethical Committee (CEEA) and the Government of Navarra (Spain).

Cell lines

The XFM (provided by Dr. Becher, Mount Sinai, New York), XFM-luc, and NP53-mCherry were cultured in DMEM supplemented with 10% fetal bovine serum and 1% streptomycin/penicillin. The UC-BL6-C7 cell line (provided by Dr. Phoenix, University of Cincinnati) was cultured in a serum-free NeuroCult™ Basal medium (Mouse&Rat, STEMCELL Technologies) supplemented with NeuroCult™ NS-A Proliferation Kit (STEMCELL Technologies), 20 ng/mL of human fibroblast growth factor (FGF) and 20 ng/mL of human epidermal growth factor (EGF). The XFM and NP53 cells were generated from a tumor induced by PDGF-β signaling and INK4A and ARF loss and p53 deficiency, respectively.[41,42] The UC-BL6-C7 was derived from a tumor generated by in-utero electroporation using a plasmid encoding for the Hist1h3bK27M, DNp53, PdgfraD842V, and Acvr1G328V mutations.[29] The generation of XFM-luc was performed by incubating the XFM cells with the supernatant of HEK293T cells containing Venus-Akaluc expressing lentiviral particles obtained upon transfection with the pLenti-PGK-Venus-Akaluc and packaging plasmids. After five days in culture with Blasticidin, XFM were sorted (FACS Aria II, BD) to isolate the GFP-positive cells. NP53-mCherry cells were obtained by stable transfection of NP53 cells (also kindly gifted by Dr. Becher) cultured in a 6-well plate ($2x10^5$ cells/well) with 4μg of the pCAG-mCherry plasmid. Following a six-day culture, mCherry-positive NP53 cells were sorted and further expanded. All cell lines were cultured in an incubator at 37°C and 5% CO2 and routinely tested to dismiss Mycoplasma infection. The tdTomato-UC-BL6-C7 cells were generated by lentiviral transduction using the supernatant of HEK293T cells containing tdTomato-expressing lentiviral particles obtained with the FUtdTW and packaging plasmids transfection.

METHOD DETAILS

Tumor cell injection and *in vivo* treatments

Balb/c, C57BL6/J, and NTV-a p53fl/fl mice were injected with 3μL of free media containing 1,000 XFM (or XFM-luc), 250,000 UC-BL6-C7 and 10,000 NP53mCherry tumor cells, respectively. Tumor cells were injected in the infratentorial area using the guide screw system as described previously.[62] Three to seven days after (depending on the tumor model), animals were intratumorally treated with 10^7 pfu of the adenovirus Delta-24-RGD and 24μg of an antibody against CD40 (clone FGK4.5; BioXcell) resuspended in 3μL of PBS. An IgG-matched antibody was used as a control (RatIgG2a, clone 2A3; BioXCell). Mice were monitored at least three times per week. The tumor growth of mice bearing the XFM-luc and UC-BL6-C7 (that expresses eGFP and luciferase) was monitored by measuring the bioluminescence five minutes upon administering 100mg/kg of Beetle Luciferin Potassium Salt (Promega) with the PhotonIMAGER Optima system (BiospaceLab). Long-term responder mice were used to study the development of immunological memory. They were intracranially re-challenged with the same cell line as the primary tumor. To assess T-cell resident memory generation, 0.5 mg/kg of FTY700 (Sigma-Aldrich) was administered daily by i.p, starting 24 hours after tumor cell injection. The blockade of CSFR1 was performed with 50 mg/kg daily oral administrations of PLX-3397 (MedChemExpress) for ten days, starting 24 hours before treatment. T lymphocytes were evaluated in blood, and microglia were assessed in the brain to evaluate the efficacy of the drugs. Animals were monitored at least three times per week.

Cell isolation and flow cytometry assays

Tumors, spleen, and cervical lymph nodes were harvested six days post-treatment. Collected tumors and lymph nodes were cut into small pieces and enzymatically disaggregated, incubating them in a digestion mix of 1mg/mL collagenase D and 40μg/mL DNAse I for 20 minutes at 37°C. Later, organs, including the spleen, were mechanically disaggregated with GentleMacs (Miltenyi) and passed through a 70-μm cell strainer in order to obtain single-cell suspensions. Tumor-infiltrating lymphocytes were isolated from dead and nervous system cells with a 37% Percoll gradient (GE Healthcare) upon a 20-minute 500g centrifugation at room temperature. RBC solution was used to remove red blood cells. Single-cell suspensions were surface stained with an antibody mix including mouse FcR-Block (BD Bioscience) for 20 min at 4°C in FACS buffer. Intracellular staining was performed with the Foxp3/Transcription Factor Staining Buffer Set (Invitrogen) according to the manufacturer's instructions. PromoFluor840 was used as a viability marker.

H&E and immunofluorescence

Paraffin-embedded sections of mouse brains were deparaffinized, hydrated, and treated with peroxidase. Slides were stained with hematoxylin and eosin to confirm the presence of the tumor. Afterward, sequential sections were incubated with a rabbit anti-mouse CD11c antibody (Cell Signalling) at 4°C overnight. After washing, the secondary antibody anti-rabbit in Alexa Fluor 647 (Thermo Fisher Scientific) was incubated at RT for 2 hours. DAPI (S7113 EMD Millipore) was used for nuclei staining. Slides were mounted using ProLongTM antifade reagent (Invitrogen). H&E images were acquired with the Aperio CS2 Digital Pathology Slide Scanner (Leica Biosystems). Immunofluorescence images were acquired in an LSM 800 confocal microscope (Zeiss). For quantification, Z stack tile scans of the whole tumor area were taken. Images were analyzed using ImageJ.

LegendPlex

Cytokine and chemokine concentration from tumors was measured with LEGENDplex mouse inflammation and mouse pro-inflammatory chemokine panels (BioLegend). Samples were acquired with the Cytoflex LX cytometer (Beckman Coulter) and analyzed with the LEGENDplex Data Analysis Software V.8.0 (Vigene Tech Inc., USA).

ELISPOT assays

For the ex-vivo stimulation assays, $2x10^5$ splenocytes from XFM-bearing mice obtained seven days after treatment with IgG control, the Delta-24-RGD, the anti-CD40, or the combination were co-cultured with $1x10^5$ RGD- or mock-infected XFM cells for 48 in a 96-well plate. The mouse IFN-γ ELISPOT set (BD Biosciences) was used according to the manufacturer's instructions. Results were measured with the Immunospot S6 Analyzer (Macro, Immunospot).

cDC1 differentiation

cDC1 were generated from bone marrow (BM) precursors from 4-week-old C57Bl/6J mice. Briefly, the tibiae and femur of mice were collected and placed on ice-cold PBS. BM precursors were isolated by flushing with PBS using a syringe and filtered in a 70um cell strainer. The solution was centrifuged at 1,200 rpm for 5 min, and red blood cells were lysed by adding ACK lysis solution. Cells were suspended in DC culture medium (RPMI 1640 media containing 0.05mM 2-mercaptoethanol, Glutamax, MEEM, HEPES, Sodium pyruvate, 10% FBS and 1%Penicillin/Streptomycin) supplemented with 200ng/ml Flt3l (BE0342, BioXCell) and 3.33 ng/ml GM-CSF (Immunotools). After replacing the medium on days three and six, non-adherent cells were collected and seeded in a new flask on day eight. On day 14, cDC1 presence was verified by flow cytometry.

In vitro DC phagocytosis, maturation, and antigen presentation assays

Ex vivo differentiated cDC1 were exposed to 10 μg/ml of CD40 antibody or isotype-matched control and cultured at a 1:3 ratio with tdTomato-expressing UC-BL6-C7 cells infected with 100 MOIs of Delta-24-RGD. The frequency of tdTomato positive DCs and their tdTomato MFI was measured by flow cytometry after four and 24 hours of co-culture to asses DC phagocytic capacity. *Ex vivo* cDC1 were incubated with the conditioned media (CM) of UC-BL6-C7 cells after being transduced with 100 MOIs Delta-24-RGD for 24 hours, in the presence of different concentrations of the CD40 antibody (10 μg/ml) or an isotype-matched control. DC activation and maturation markers, such as IA/IE, CD80, CD86, PDL1, and TNFα, were measured by flow cytometry 24 hours later. Mature cDC1 were pulsed with 1μg/ml of gp100 for three hours, washed with PBS, and plated in a 1:3 ratio with VPD450 dye (BD Bioscience)-labelled hgp100 TCR-specific mouse CD8 T cells (isolated from PMEL mice splenocytes). In addition to T cell proliferation, activation markers such as Granzyme B and IFNγ were measured by flow cytometry 48 hours later.

RNAseq

After six days of treatment, total RNA was isolated from 24 XFM tumors (six tumors per treatment group) using the RNeasy® Mini Kit (Qiagen) according to the manufacturer's instructions. The RNA concentration and quality were measured using a Qubit HS RNA Assay Kit (Thermo Fisher Scientific) and 4200 TapeStation with High Sensitivity RNA ScreenTape (Agilent Technologies). RNA samples showed good quality, with RIN values ranging from 7.6 to 8.2. Subsequently, library preparation was performed with 100 ng of total RNA using the Illumina Stranded mRNA Prep Ligation kit (Illumina) following the manufacturer's instructions. RNA-Seq Analysis. Quality assessment of the raw sequencing reads was performed using FastQC (v0.11.8) to evaluate data integrity. Trimming of low-quality bases and adapter sequences removal were achieved through Trimmomatic (v0.38). The alignment of the high-quality reads to the Mus musculus reference genome (GRC39, version 27) was conducted using STAR (v2.7.0). Transcript quantification was executed with the featureCounts function from the Subread (v1.6.3). Gene expression data is available at the GEO repository (GSE292938 accession number).

QUANTIFICATION AND STATISTICAL ANALYSES

Differences in immune cell frequencies, tumor growth, and mice survival were analyzed with GraphPad Prism (GraphPad Software, La Jolla, CA). Differential expression analysis was implemented utilizing the edgeR (v3.40.2) R package. Gene enrichment and functional annotation were performed using the clusterProfiler R package (v4.6.2). P values <0.05 were considered significant. The outliers have been removed from the statistical analyses. ns, p>0.05; *p<0.05; **p<0.01; ***p<0.001; ****p<0.0001.

Cell Reports Medicine, Volume *6*

Supplemental information

Targeting the CD40 costimulatory receptor to improve virotherapy efficacy in diffuse midline gliomas

Sara Labiano, Javier Marco-Sanz, Iker Ausejo-Mauleon, Virginia Laspidea, Reyes Hernández-Osuna, Marc Garcia-Moure, Daniel de la Nava, Sara Nuin, Marisol Gonzalez-Huarriz, Timothy N. Phoenix, Ibon Tamayo, Marta Zalacain, Andrea Lacalle, Lucía Marrodan, Montserrat Puigdelloses, Irati Hervás-Corpión, Maria C. Ochoa, Noelia Casares, Oren J. Becher, Candelaria Gomez-Manzano, Juan Fueyo, Jaime Gallego Perez-Larraya, Ana Patiño-Garcia, and Marta M. Alonso

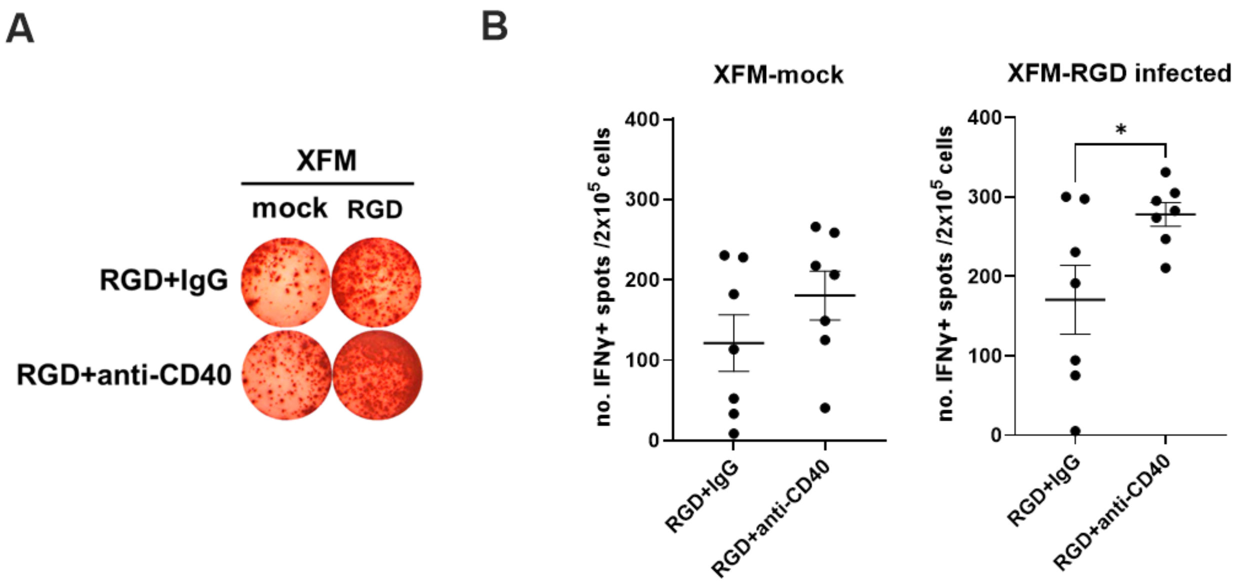

Figure S1: The anti-CD40 agonist enhances the Delta-24-RGD-specific adaptive immune response. Related to **Figure 2.**

(A) Representative picture of the wells from the ELISpot plate co-culturing RGD- or mock-infected XFM cells with splenocytes isolated from XFM-bearing mice at day six after being treated with the virus alone or the combination. (B) Analyses of IFNγ-producing splenocytes from the indicated groups measured by ELISpot 72h upon co-culturing with XFM cells. Paired T-test and Wilcoxon test were used for statistical analysis of the ELISpot. n =6-7 samples per group. ($p<0.05$*)

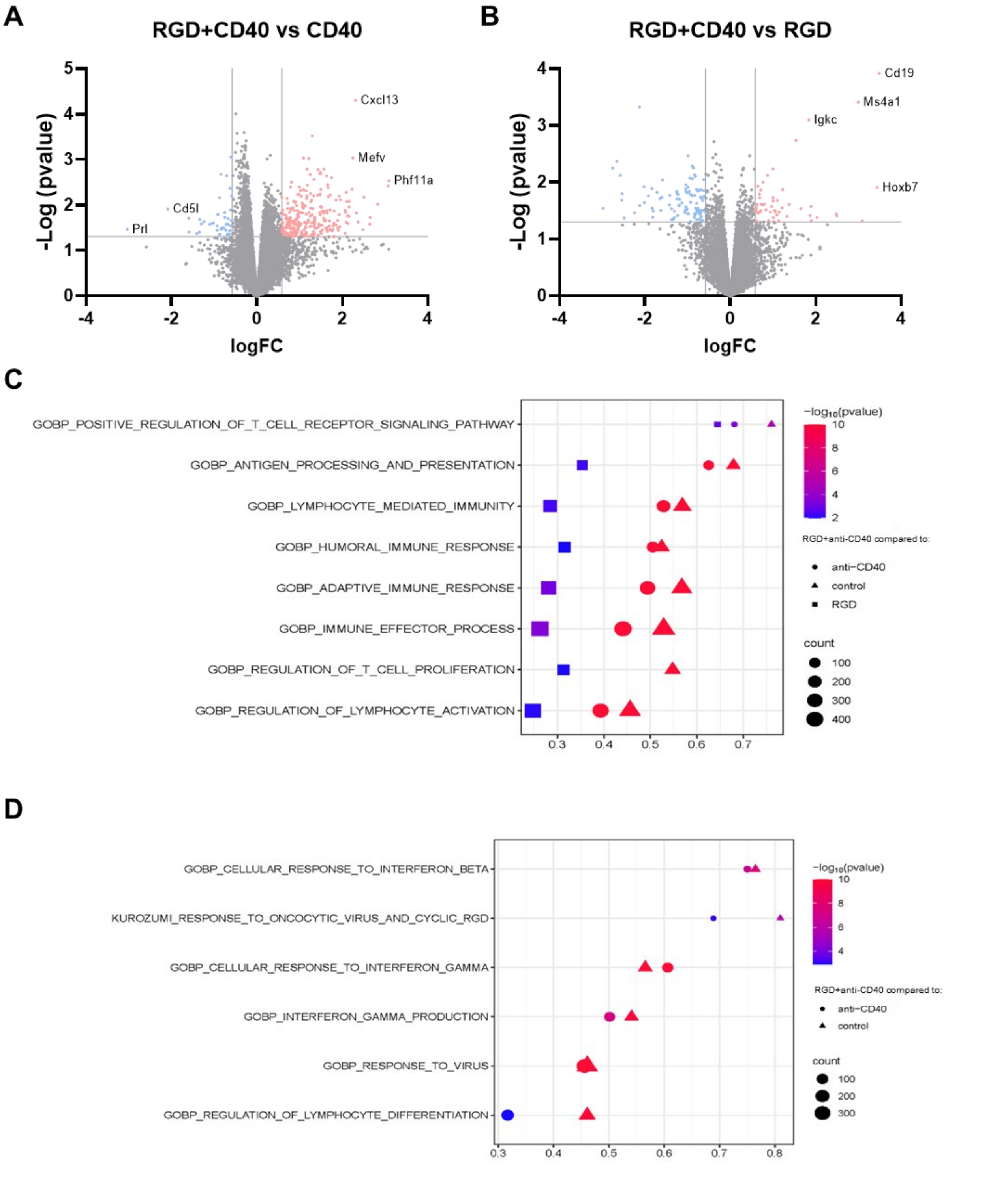

Figure S2. Transcriptional enrichment in immune-related genes and pathways that involve immune response in tumors treated with RGD+anti-CD40. Related to Figure 4.
(A-B) Volcano plots showing differentially expressed genes (DEG) from RNAseq of the bulk XFM tumors treated with the combination and compared to anti-CD40 or Delta-24-RGD alone at day 6. (C-D) Gene Set Enrichment Analyses within the combination and the indicated group of treatments, showing immune-related pathways expressed in the tumors at day 6 post-therapy. T-tests were used for statistical analyses. n =6 samples per group of treatment.

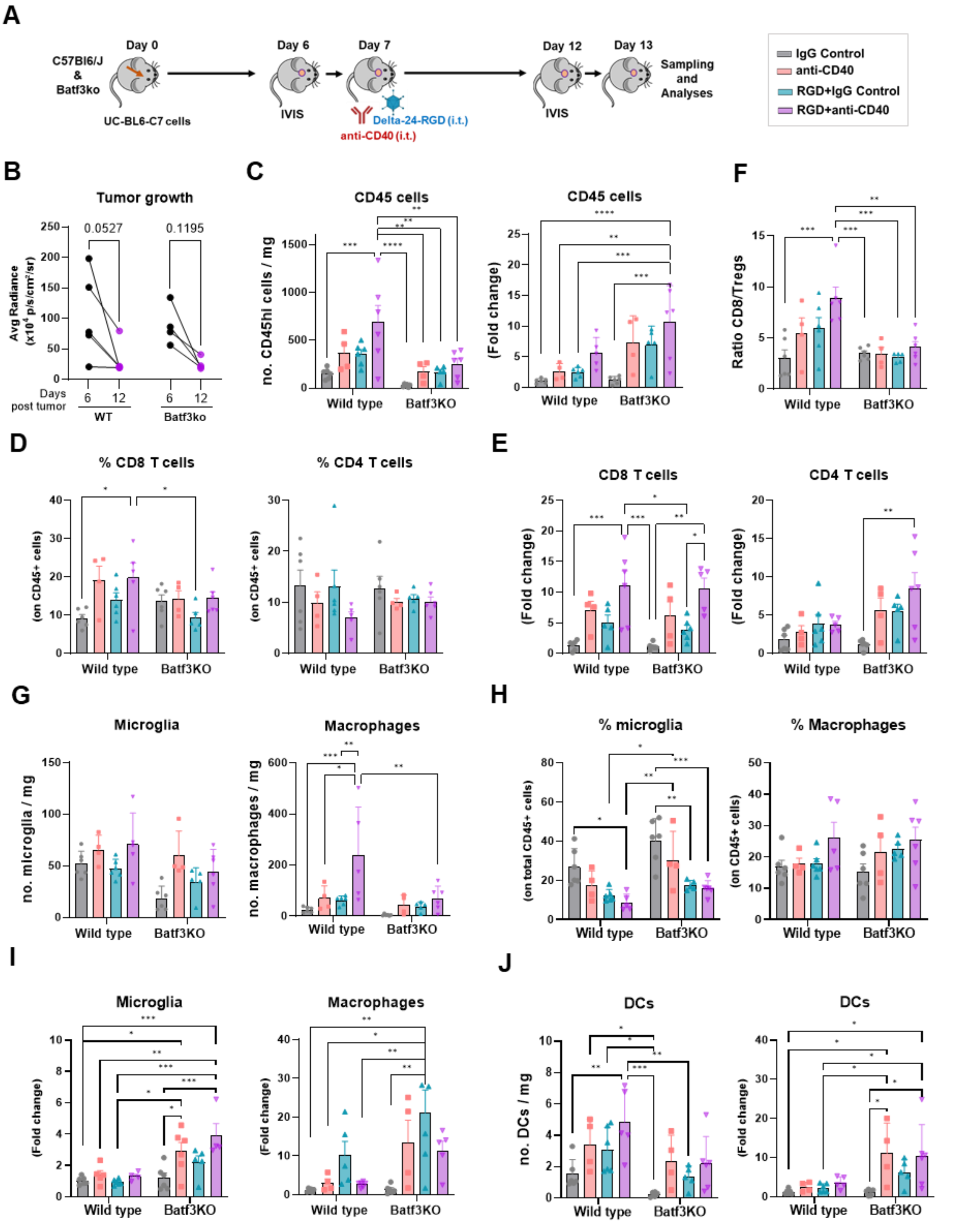

Figure S3. Tumor immune response UC-BL6-C7-bearing Batf3KO mice after the combination therapy. Related to Figure 4.

(A) Schedule of the experimental procedures performed in UC-BL6-C7-bearing C57Bl6/J and Batf3KO mice to study the immune response by flow cytometry. (B) Tumor growth decrease five days after treatment with the combination in wild type and Batf3KO mice. (C) Numbers of the immune infiltrating cells (CD45 high expressing cells) per tumor mg, and their fold change related to the mean of the respective IgG control groups. (D) Frequencies of CD8 and CD4 T

lymphocytes per mg of tumor. (E) Fold-change of CD8 and CD4 T cells in the indicated treatment related to the mean of the respective IgG control groups. (F) CD8/Tregs ratio measured in the tumor. (G-I) Numbers of microglia (CD45loCD11b+) and macrophages (CD45hiCD11b+) per mg, their frequency, and fold-change from UC-BL6-C7-tumors. (J) Numbers of DCs per tumor mg and DCs fold-change at day six post-treatment. Two-way ANOVA test was used for statistical analyses. n =4-6 samples per group. (p<0.05*; p<0.01**; p<0.001***; p<0.0001***)

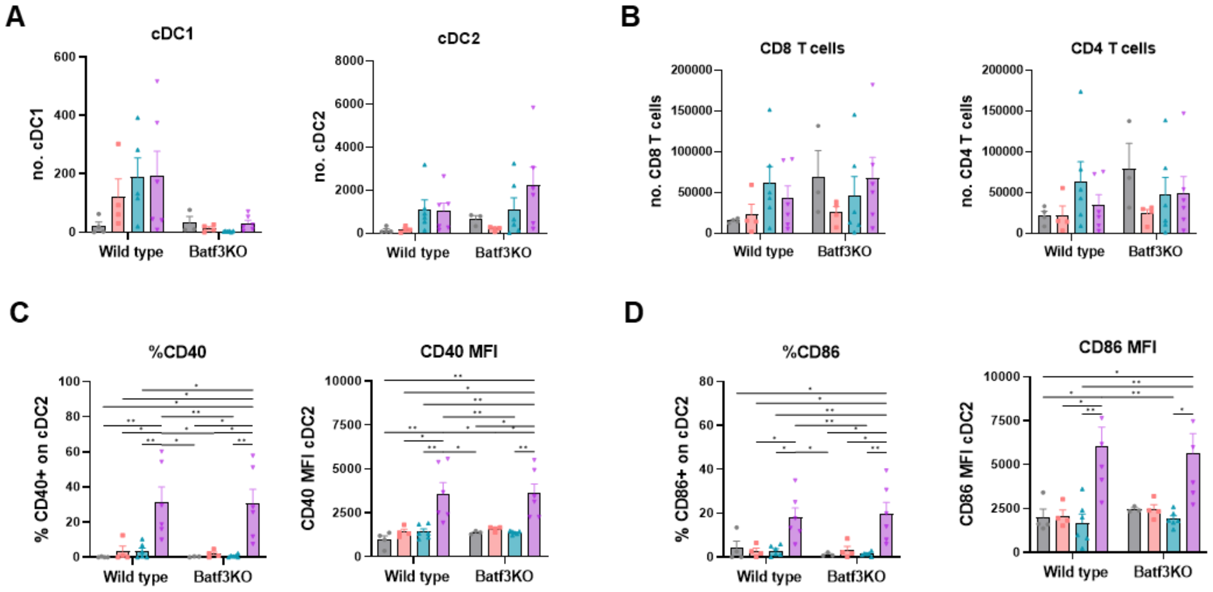

Figure S4. Batf3ko mice show similar cDC2 activation and T cell numbers in the draining-lymph node as wild type mice. **Related to Figure 4.**

(A and B) Absolute numbers of conventional DC (cDC1 and cDC2) and T lymphocytes (CD8 and CD4 T cells) obtained from deep cervical lymph nodes six days following the indicated treatments. (C and D) Frequency and expression (as Mean of Fluorescence Intensity, MFI) of CD40 and CD86 co-stimulatory receptors on cDC2 from draining lymph nodes in wild type and Batf3ko mice. Two-way ANOVA test was used for statistical analyses. n =4-6 samples per group. (p<0.05*; p<0.01**; p<0.001***; p<0.0001***)

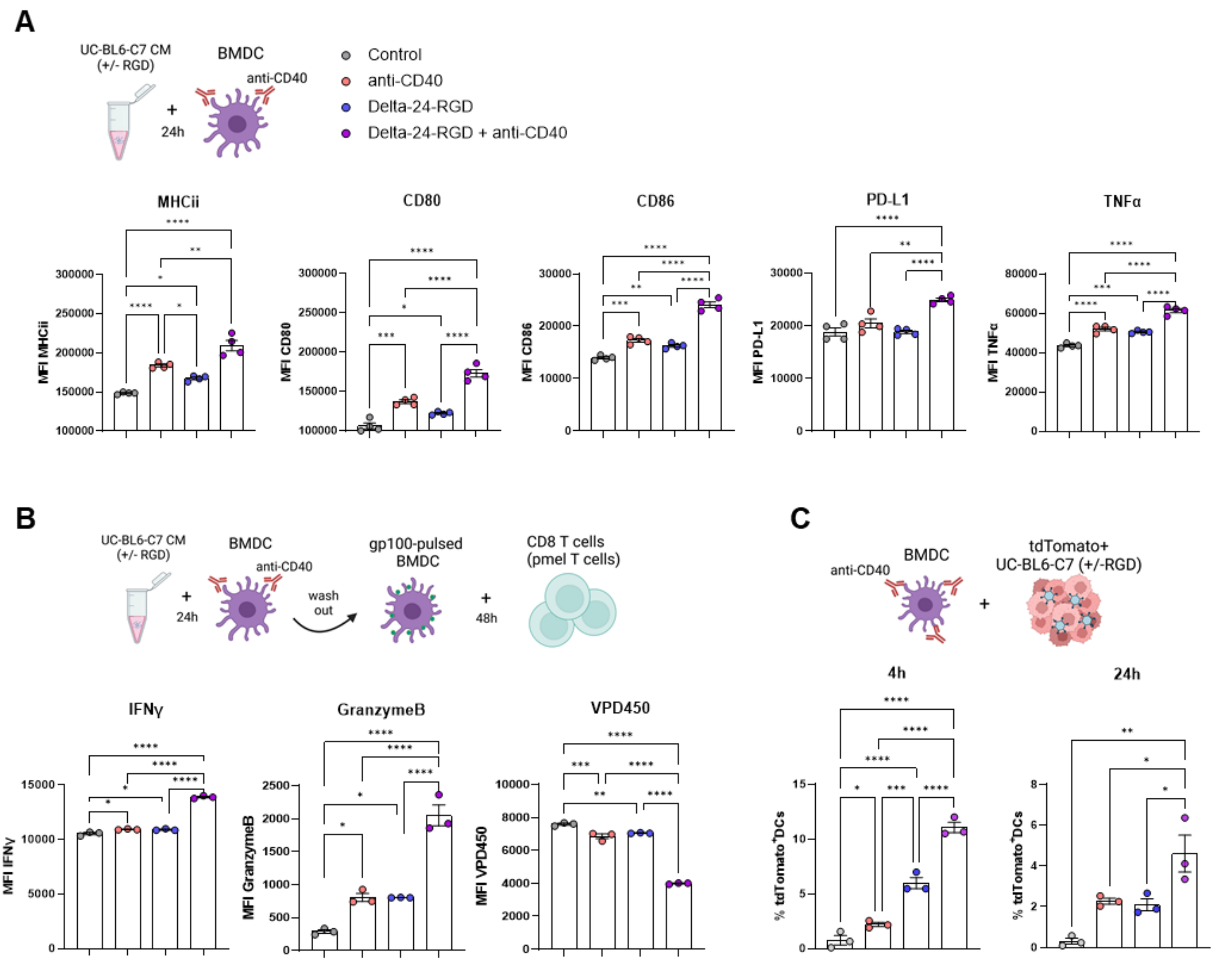

Figure S5. The Delta-24-RGD and the anti-CD40 agonist synergize in DC maturation, antigen presentation, and tumor phagocytic capability. Related to Figure 4.

(A) In vitro maturation assay of bone marrow-derived type 1 DCs (BMDC) incubated with the supernatant of RGD-transduced UC-BL6-C7 cells in the presence of IgG control or the anti-CD40 agonist antibody. Expression of MHC class II, CD80, CD86, and PDL1 maturation markers and the production of TNFα were assessed in cDC1 cells by flow cytometry 24 hours later. (B) Antigen presentation assay to pmel CD8 T cells with mature BMDC from A pulsed with the gp100 peptide. Graphs show the production of T cell activation (IFNγ) and cytotoxic markers (Granzyme B), and the proliferative capacity of CD8 T cells (decrease in VP450 cell dye) measured by flow cytometry 48 hours after. (C) Percentage of BMDC positive for tdTomato four and 24 hours after being cocultured with tdTomato+ UC-BL6-C7 transduced or not with the RGD virus. MFI: mean of fluorescence intensity. One-way ANOVA and the Kruskal-Wallis test were used for statistical analyses. n =4-6 samples per group. (p<0.05*; p<0.01**; p<0.001***)

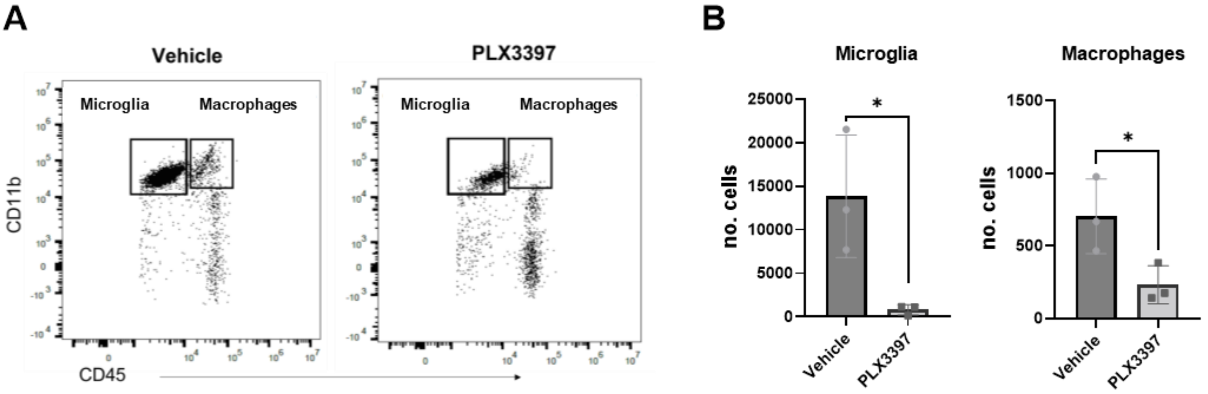

Figure S6. PLX3397 reduces the number of microglia and macrophages the tumor. Related to Figure 5.
(A) Representative example of microglia ($CD45^{lo}CD11b^{+}$) and macrophages ($CD45^{hi}CD11b^{+}$) from XFM-tumors three days upon starting the treatment with 50mg/kg of PLX3397 or the vehicle. (B) Quantification of the number of microglia and macrophages present in the tumors. The Mann–Whitney test was used for a number of cell analyses (n = 3 mice per group). ($p<0.05$*)

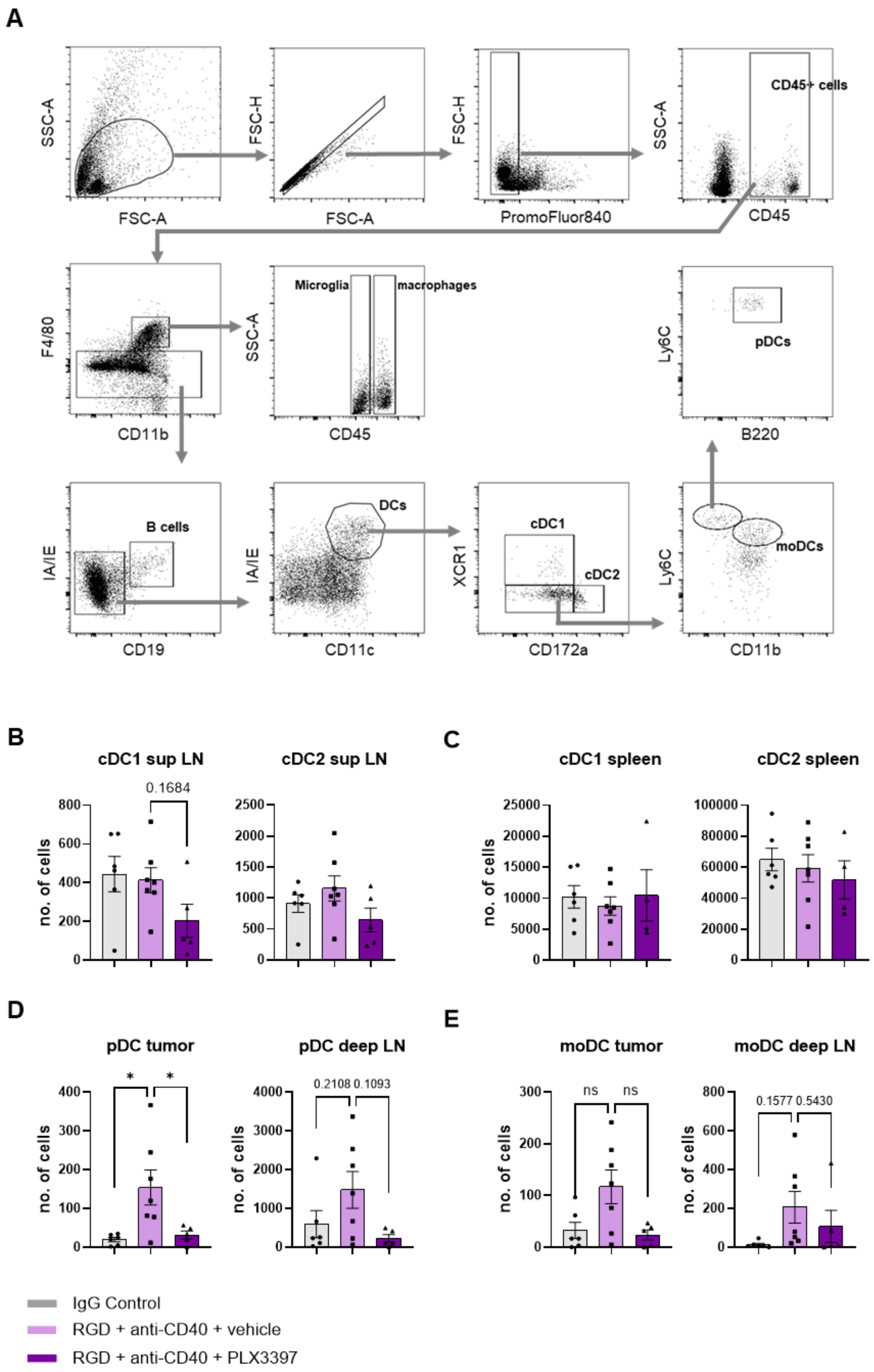

Figure S7. Effect of the inhibition of CSF1R in the recruitment of different types of DCs. Related to Figure 5. (A) Gating strategy followed to analyze the infiltration of the indicated DCs types in the tumor, lymph nodes and spleen. (B-C) Flow cytometry analyses of cDC1 and cDC2 infiltration of XFM-bearing mice in the superficial non-draining lymph nodes and the spleen six days post-treatment. (D-E) Numbers of monocytic DCs (moDCs) and plasmacitoid DCs (pDCs) in the tumor and deep draining lymph nodes 6 days post-treatment. One-way ANOVA and the Kruskal-Wallis test were used for statistical analyses from C-F data. n =6-7 samples per group. ($p<0.05$*)
